# Uncrosslinked Thermoresponsive Hybrid Magnetic Nanospheres Directly Prepared from Poly(*N*‑isopropylacrylamide) that Behave as Heating Rate Nanosensors

**DOI:** 10.1021/acs.biomac.5c01603

**Published:** 2025-10-17

**Authors:** María García-Maestre, Laura Cervera-Gabalda, Eva Natividad

**Affiliations:** Instituto de Nanociencia y Materiales de Aragón (INMA), 82976 CSIC-Universidad de Zaragoza Campus Río Ebro, María de Luna, 3, 50018 Zaragoza, Spain

## Abstract

Poly­(*N*-isopropylacrylamide) (PNIPAM) provides thermoresponsiveness to nanoobjects containing magnetic/plasmonic nanoparticles (NPs) for biosensing and biomedicine. Conjugation methods include the grafting of PNIPAM onto NPs or the embedding of NPs in PNIPAM nanogels. Nanoobjects are often obtained simultaneously with monomer (NIPAM) polymerization, and the cytotoxicity of unreacted NIPAM represents a non-negligible concern. Herein, a facile and versatile miniemulsion method employing already polymerized PNIPAM is developed. Miniemulsion is achieved through PNIPAM globulization above the lower critical solution temperature (LCST) and stabilized by poly­(vinyl alcohol) (PVA). Aqueous decants, obtained after solvent evaporation, contain thermoresponsive nanospheres with PNIPAM/PVA blends, stable in water for months, monomodal in size at high magnetic NP contents, biocompatible and with hyperthermia capability. Unlike the shrinking/swelling displayed by PNIPAM-based nanogels, these uncrosslinked nanospheres disintegrate/rebuild, with a size after disintegration undoubtedly related to their heating rate across LCST, behaving as unprecedented dual hyperthermia agents and heating rate nanosensors without further sensing molecules.

## Graphical abstract

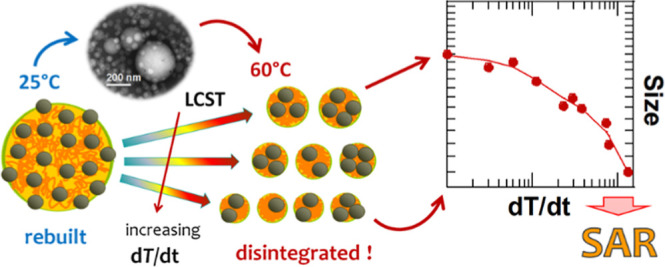

## 1 Introduction

Materials are considered smart when they exhibit properties that can change in a significant, dynamic, reversible and controlled manner against slight variations in external conditions. In particular, smart materials are able to respond to external stimuli such as temperature, pH, light, pressure, electric or magnetic fields, and the presence or concentration of chemical or biological species, through changes in shape, size, phase, solubility, assembly or optical properties. This remarkable ability enables these materials to perform significant functions as sensors,^1^ actuators,^2^ drug release mediators,^3^ catalyst,^4^ bioseparators,^5^ etc.

Temperature is one of the most relevant and studied stimuli in polymers and has direct implications for sensing and temperature-driven processes, especially in biological applications. A wide variety of thermoresponsive polymers, such as poly­(*N*-vinylcaprolactam)^6^ and several poly­(acrylamide)­s,^1^ are available. However, poly­(*N*-isopropylacrylamide) (PNIPAM) is no doubt the most used, since it is biocompatible and has a lower critical solution temperature (LCST) of ca. 32 °C, slightly lower than body temperature, but adjustable through copolymerization of NIPAM with other hydrophilic or hydrophobic monomers. Below the LCST, PNIPAM is soluble in water, and the chains adopt a hydrated coil configuration. Readily above the LCST, PNIPAM becomes hydrophobic, expels water molecules and acquires globular arrangements, undergoing the so-called coil-to-globule transition. This transition can be detected, for instance, by measuring turbidity or by determining the size variation of polymer entities due to coil-to-globule changes.

Thermoresponsiveness is achieved not only by using free PNIPAM chains in solution but also by using more convenient configurations. PNIPAM chains can be grafted onto surfaces to stretch and retract across the LCST, thus allowing the switching of, e.g., surface wettability.^7^ Additionally, modifying PNIPAM chains through copolymerization or functionalization with, for example, fluorescent dyes, allows accurate optical thermometers to be obtained in a well-established temperature range.^8^ These polymers are obtained mainly by common free radical polymerization or by reversible addition–fragmentation chain-transfer (RAFT) polymerization.^9^ Crosslinked gels and nanogels, in which PNIPAM forms networks capable of absorbing large quantities of water without dissolving, can swallow and shrink across the LCST, triggering processes such as drug release while retaining integrity in the biological environment. These gels are usually obtained by free radical polymerization of NIPAM in the presence of a crosslinker, although more specific methods, such as miniemulsion polymerization, have been reported.^10^ Thermoresponsive hybrid materials can also be developed, for example, including magnetic or plasmonic inorganic nanoparticles (NPs) to tackle biomedical applications like heat-assisted drug release, as well as other functionalities, including plasmonic or magnetic hyperthermia,^11,12^ and different biosensing applications (temperature, bacterial detection, medical diagnosis, etc.).^1^

Two approaches are commonly used to conjugate PNIPAM and magnetic or plasmonic NPs, one as inorganic cores with polymeric shells, and another as inorganic nanoparticles embedded in/on polymeric spheres, often in the form of gels. Bioapplications tackled with core–shell structures are, for example: (i) thermoresponsive barriers for drug release,^13^ in which PNIPAM copolymer chains are simultaneously formed and grafted onto iron oxide core/mesoporous silica shell nanoparticles through radical polymerization of the comonomers; (ii) optical temperature sensing, where the functionalization via grafting of Au NPs^14^ with PNIPAM (RAFT polymerization) produce changes in the optical properties of the Au NPs. Similar bioapplications were undertook embedding NPs in PNIPAM, using, for example: (i) PNIPAM/Fe_3_O_4_–ZnS hollow spheres,^15^ in which PNIPAM nanogels were first synthesized from NIPAM and a crosslinker, after which Fe_3_O_4_ and ZnS nanoparticles were precipitated in situ on the nanogel surface, giving rise to entities that integrate magnetism, luminescence (for tracking drug release) and temperature response; (ii) Au NPs in PNIPAM-based crosslinked microgels, prepared by mixing microgels and AuNP solutions, in which the shrink/swell behavior of PNIPAM induced changes in the Au NP arrangement and plasmon modes, leading to optical determination of temperature changes.^16^ Eventually, both approaches can be combined for the same nanomaterial to incorporate magnetic and plasmonic nanoparticles.^17^

A common feature of the abovementioned methods for obtaining PNIPAM-based materials is that polymerization of the monomer (NIPAM) often occurs simultaneously with material preparation, either to enable grafting (“grafting-from” processes^18^) or crosslinking. In biomedical applications, one concern about this approach is the cytotoxicity of unreacted NIPAM, which could be difficult to eradicate, especially in hydrogels, due to its water solubility. This concern was one of the starting motivations of this work. Typical techniques for monomer elimination include precipitation, dialysis, or centrifugation, albeit they can modify the physical properties of the suspensions.^19^ We thus aimed to obtain thermoresponsive hybrid magnetic nanospheres with a novel NP embedding approach, through a preparation process that started directly from PNIPAM, allowing prior analysis and purification of the polymer. Another incentive was the possibility of attaining a facile preparation method, which led us to select miniemulsion and solvent evaporation techniques. These are versatile processes that are widely used to conjugate diverse biocompatible polymers such as polylactic acid (PLA),^20^ poly­(lactic-*co*-glycolic acid) (PLGA)^21^ or polycaprolactone (PCL)^22^ with NPs but, notably, they are not used with PNIPAM (as polymer). A third stimulus to this work, related with the absence of chemical crosslinking, was the prospect of finding unprecedent behaviors and functionalities of the nanospheres, as a consequence of bringing into play different thermal response from that of nanogels.

In this paper, we first described the successful preparation method developed. Next, we devoted several sections to understanding different aspects of the method, as well as the role of each component of our hybrid nanospheres. For this purpose, we analyzed, either pure starting solutions, or selected nanosphere suspensions covering diverse stages of the process and different compositions/quantities of polymers and magnetic nanoparticles. Finally, we displayed the characterization and evaluation of key functionalities of the uncrosslinked nanospheres obtained. In particular, these nanospheres have emerged as dual hyperthermia agents and reversible heating rate nanosensors since heating rates across the LCST can be directly deduced from the nanosphere size above the LCST. To the best of our knowledge, no similar nanosensors have been reported previously, and these findings open the way to a novel approach for determining the heating ability of nanoobjects suitable for magnetic hyperthermia, with information obtained directly from the nanoobject and without the use of foreign molecules.

## 2 Materials and Methods

### 2.1 Materials

Nanospheres were prepared using solvents, polymers and iron oxide magnetic nanoparticles (NPs). The solvents used were distilled water and reagent grade chloroform purchased and used as received from Sigma-Aldrich. The polymers used were poly­(*N*-isopropylacrylamide) (PNIPAM, MW 20–40k), poly­(vinyl alcohol)-hydrolyzed 87–89% (PVA, MW 13–23k), and poly­(d,l-lactide-coglycolide) 50:50 (PLGA, MW 30–60k) purchased and used as received from Sigma-Aldrich. Two different types of NPs, NP1 and NP2, with distinct morphologies and sizes were employed (see Figure S1 for details). NP1 NPs were synthesized in the laboratory^23^ through an organic phase thermal decomposition method in which magnetite nanoparticles of approximately 6 nm were first produced and then subjected to 27 growth steps. As a result, faceted and/or twinned NPs were obtained with a TEM size of 15 ± 3 nm. NP2 NPs were purchased from Ocean NanoTech, LLC (SOR-20 iron oxide NPs in chloroform) and had a TEM size of 21 ± 2 nm. Both NP1 and NP2 were stabilized with oleic acid (NP1 additionally with oleylamine), and the nominal concentrations of the suspensions were 2 and 25 mg/mL, respectively. The aim of using two distinct NPs was to determine whether different NP shapes, sizes and concentrations could be used for the preparation of our hybrid nanospheres.

### 2.2 Preparation of Nanospheres

We modified an existing method aimed at obtaining PLGA/PVA nanospheres^20,21^ loaded with NPs. We first fabricated 3P (PNIPAM/PLGA/PVA) samples without, with NP1 and with NP2 nanoparticles (3P-noNP, 3P-NP1 and 3P-NP2, respectively). Typically, for samples with NPs, the process started by evaporating the solvent (200 μL (NP1) or 400 μL (NP2)) of the NP suspension and resuspending the NPs in 5 mL of CHCl_3_. For samples without NPs, only CHCl_3_ was used. Afterward, 7.5 mg of PLGA and 42.5 mg of PNIPAM were added and dissolved by stirring. This organic solution was then covered, heated to ca. 60 °C and poured into 40 mL of a previously heated PVA aqueous solution (7.5 mg/mL). The mixture was covered to avoid evaporation of chloroform or water and stirred at ca. 60 °C for 1 h. Afterward, the organic solvent (CHCl_3_) was allowed to evaporate overnight while stirring at ca. 60 °C. Finally, distilled water was added to bring the volume to approximately 45 mL. This preparation was called the RAW sample. For separation by decantation, 40 mL of RAW sample was poured into a 100 mL separating funnel together with 32 mL of chloroform, shaken and left to stand for a short period of time (approximately 5 min, until two phases were clearly distinguished). The aqueous part (top fraction) was removed directly with a Pasteur pipet and was called the DEC–AC sample. To ensure evaporation of the chloroform dissolved in water, the aqueous decant was left overnight with mechanical stirring at room temperature. The organic part (bottom fraction) was poured from the funnel into a beaker with 160 mL of distilled water at ca. 60 °C with mechanical stirring and left until the chloroform was fully evaporated (typically overnight). The evaporated water was replaced at the end. Once transferred to water, this sample was called the DEC–ORG sample and was ready to be characterized similarly to the DEC–AC one. Particularly for samples with NP2, separation by decantation was performed twice, i.e., sample 3P-NP2-DEC-AC was subjected to decantation similar to that of 3P-NP2-RAW. Eventually, all the above processes were repeated without PLGA, i.e., completely replacing PLGA with PNIPAM, and the samples were labeled 2P (PNIPAM/PVA). In addition, for this system, five different 2P-NP2 samples were prepared using PVA solutions with varying concentrations, in particular using 25, 50, 100, 200 and 400% of the nominal concentration, i.e., 7.5 mg/mL. A complete list with all these acronyms is included in the Supporting Information.

### 2.3 Dynamic Light Scattering

DLS was performed using a Zetasizer Nano ZS from Malvern Panalytical. DEC-ORG samples were measured with their original concentration, while RAW and DEC–AC ones were diluted to half of their concentration. Hydrodynamic diameters were measured in the 25–60 °C temperature range by placing 1 mL of suspension or solution in a PS cuvette. The temperature was equilibrated for 30 s before conducting a series of 5 measurements with a 10 s interval between each. Typically, between 3 and 5 series were averaged for each determination, returning values of intensity (*I*) versus size (*d*). Volume and number distributions were calculated from the intensity one using Rayleigh’s scattering law as the normalized *I*/*d* ^3^ and *I*/*d* ^6^ functions, respectively. These distributions serve two purposes. First, the volume provides a more balanced view of multimodal suspensions since the intensity emphasizes larger particles, while the number highlights smaller particles. Second, number distributions are the nearest to microscopy results, i.e., to real sizes. In any case, since the diameters obtained by DLS are hydrodynamic, they will always be somewhat larger than real ones. All distributions were fitted to lognormal distributions as *y* = *C*_LN_·(*d*·σ_LN_·(2·π)^1/2^)^−1^·exp­[−(ln­(*d*)-ln­(*d*_LN_))^2^/(2·σ_LN_^2^)], where *y* stands for intensity/volume/number counts, *C*_LN_·is a constant, ln­(*d*_LN_) is the average of the logarithmic values and σ_LN_ is the standard deviation of the logarithmic values. The average sizes, *d*_m_, were calculated from these parameters as *d*_m_ = *d*_LN_·exp­[σ_LN_^2^/2], and the standard deviations, σ, were calculated as σ = *d*_LN_^2^·exp­(σ_LN_^2^)·[exp­(σ_LN_^2^) – 1]. The zeta potentials (ZPs) of the suspensions and solutions were determined using the same equipment. One milliliter of each sample was placed in a DTS 1070 cuvette following the instructions of the manufacturer. Measurements were made at 25 and 60 °C, previously equilibrating the temperature for 30 s, and taking three measurements with a rest time of 10 s. Mean values and standard deviations were obtained using Zetasizer Software.

### 2.4 Transmission Electron Microscopy

TEM was conducted on a JEOL JEM 1010 instrument at an acceleration voltage of 100 kV. The samples were negatively stained with phosphotungstic acid (PTA). Typically, carbon-coated copper grids were first covered with 10 μL of PTA contrast agent (20 mg of PTA + 2 mL of distilled water + 90 μL of 0.5 M NaOH), loaded with 10 μL of sample, and finally left to dry. This process was performed twice per sample, once below the LCTS of PNIPAM (at room temperature) and once above it (heating the PTA contrast, samples and grids on a plate at 70 °C). Histograms were obtained measuring the diameter (size) of more than 300 nanospheres, and the resulting data were fitted to normal (Gaussian) distributions to derive mean values and standard deviations. Cryo-TEM was performed using a TECNAI T20 SuperTwin (FEI-Thermo Fisher) at 200 kV. Vitrification of the sample was performed with an FEI Vitrobot Mark IV. Namely, a 3 μL drop was placed on a copper grid (300 mesh Quantifoil, hydrophilized by glow-discharge treatment just prior to use) within the environmental chamber of the Vitrobot. Excess water was first removed, and then the grid was immersed in liquid ethane. The vitrified sample was transferred through the 655 Turbo Pumping Station (Gatan) to a 626 DH Single Tilt Cryo-Holder (Gatan), where it was handled and analyzed at 100 K.

### 2.5 Dry Residue

The concentrations of the RAW, DEC–AC and DEC–ORG suspensions were determined from dry residues. Typically, 1 mL of vortexed sample was extracted and weighed on a standard aluminum weighing boat before and after water evaporation on a hot plate at 70 °C. The mass of the remaining material (polymers and NPs) was used to calculate mass concentrations in mg/mL. From concentrations and volumes, the total RAW mass decanted could be estimated, as well as the total mass present in DEC–AC and DEC–ORG decants. This approach allowed us to calculate which percentage of the RAW sample remained in the aqueous or organic decant solution and how much was lost during the process.

### 2.6 Quantification of Amount of Magnetic Material

A combination of elemental analysis and magnetic measurements was used to quantify the amount of magnetic material trapped in the nanospheres. Dry aliquots of NP1 and NP2 nanoparticles (iron oxides + oleic acid) were first weighed and then subjected to magnetization measurements against a static field, *M*(*H*), at 300 K and with field values between 0 and 5 T using superconducting quantum interference device (SQUID) magnetometers from Quantum Design. First, the total magnetic moment, μ (emu), was obtained. Once all the characterizations were performed, both aliquots were acid digested in aqua regia (HCI (≥37%) and HNO_3_ (≥65%) mixed 3:1 in volume) at 90 °C for 1 h. Distilled water was then added until a known volume was reached, providing an estimated Fe concentration between 1 and 30 ppm. Inductively coupled plasma–optical emission spectrometry (ICP–OES), conducted with an Xpectroblue-EOP-TI FMT26 (Spectro), was used to determine the real Fe content of the dry aliquots. From these data, the total mass of magnetic material could be calculated by the stoichiometry of the iron compound present. For NP1, the iron compound was most likely Fe_3_O_4_.^23^ For NP2, the supplier did not specify the type of oxide present. However, the errors associated with considering either Fe_2_O_3_ (70% in mass of Fe) or Fe_3_O_4_ (72% in mass of Fe) when calculating the mass of magnetic material are not too large. For this reason, and based on previously acquired batches, we assumed magnetite to be the iron compound present in both kinds of NPs. Finally, the saturation magnetizations, *M*_S_ (emu/g), of NP1 and NP2 were calculated by dividing their saturation magnetic moment, μ_*S*_, by the mass of the magnetic material (see Figure S2). Eventually, *M*(*H*) measurements were also performed on freeze-dried nanosphere samples, and the mass of the magnetic material was calculated by dividing the saturation magnetic moment by the *M*_*S*_ value of the NPs they contained. For the determination of μ_S_, linear μ­(*H*) trends assigned to para/diamagnetic contributions were subtracted.

### 2.7 Differential Scanning Calorimetry

DSC was performed using a Q1000 device from TA Instruments with sapphire as the calibrant for quantifying heat capacity (*C*_p_). Solid polymer and freeze-dried nanosphere samples were weighed and measured. Typically, two heating/cooling cycles covering the −50 to 200 °C temperature interval were recorded at 10 °C/min. The first heating, in which excess water evaporation due to PVA is predominant, was discarded.

### 2.8 Heating Ability

The heating ability under the application of alternating magnetic fields was determined through the specific absorption rate (SAR). When using calorimetric methods, SAR (W/g_NP_) is calculated using the formula

1 $$SAR=\frac{C}{m_{NP}}\times \left[heating\;rate\;at\;zero\;losses\right]$$

where *C* (J/°C) is the total heat capacity of the sample (+container, if any) and *m*_NP_ (g) is the mass of magnetic material. The determination of the heating rate at zero losses (°C/s) depends on the setup and method. We used a special-purpose adiabatic magnetothermal setup^24^ and the pulse-heating method,^25^ which allows calculating the heating rate at zero losses as

2 $$heating\;rate\;at\;zero\;losses=\frac{\Delta T}{\Delta t}$$

where Δ*T* is the temperature increment of the sample during the time interval of magnetic field application, Δ*t*. The use of adiabatic conditions assures that Δ*T* is measured with zero losses at any temperature. Temperature increments as a function of temperature, Δ*T*(*T*), were measured in the range 5–65 °C, using a field frequency *f* = 83 kHz, field amplitudes *H*_0_ = 2.8–8.4 kA/m, and the same dry or freeze-dried sample aliquots previously used for magnetic measurements. Additionally, for nanospheres, Δ*T*(*T*) was measured also at *f* = 125/209/295 kHz with *H*_0_ = 8.4 kA/m. The SAR was subsequently calculated from these data. The intrinsic loss parameter (ILP) (nH·m^2^/kg) was also obtained as ILP = 10^3^·SAR/(*f*·*H*_0_^2^), with the SAR in W/g, *f* in kHz and *H*_0_ in kA/m.

### 2.9 In Vitro Assays

They were performed in cell cultures of immortalized human gingival fibroblasts—hTERT (Cat. Number: T0026; Lot Number: 0089825329003; Applied Biological Materials Inc., Richmond, BC, Canada). The cells were seeded in 96-well plates at a density of 7 × 10^3^ cells/well and cultured under standard conditions at 37 °C in a 5% CO_2_ atmosphere with saturating humidity. The culture media used were low-glucose Dulbecco’s modified Eagle medium (Biowest) and Ham’s F12 (Biowest) at a 2:1 ratio supplemented with 5% (V/V) fetal bovine serum (Biowest, embryonic stem cells tested), 100 μg/mL penicillin and 100 μg/mL streptomycin (Biowest) supplemented with 50 μg/mL ascorbic acid (Sigma-Aldrich). When confluence was reached, the cells were either left untreated or treated with (i) a nanosphere suspension (0.5 mg/mL) dissolved in culture medium and previously heated for 15 min at 37 °C to ensure proper transition above the LCST before incubating with the cells; (ii) a sample of distilled water used for the fabrication of nanospheres; or (iii) 0.1% Triton-X100 (a nonionic surfactant). Cytotoxicity was assessed by the lactate dehydrogenase (LDH) test. LDH is a cytosolic enzyme that is rapidly released into the cell culture medium upon damage to the plasma membrane. Therefore, high levels of LDH activity are indicative of cytotoxicity. LDH detection in the supernatants was performed using a commercial colorimetric enzymatic assay after 30 min of incubating 50 μL of culture medium with 50 μL of reaction mixture at room temperature, according to the manufacturer’s instructions (Cytotoxicity Detection Kit, Roche Diagnostics, Mannheim, Germany). Metabolic activity was determined by the resazurin assay. Briefly, healthy cells with active metabolism can reduce resazurin to resorufin, resulting in a colorimetric change. Total metabolic activity was assessed after 24 h of treatment. For this purpose, the cells were incubated with 10 μL of PrestoBlue (Life Technologies, Carlsbad, CA, USA) in 100 μL of culture medium. Colorimetric changes were measured after 1 h of incubation at 37 °C. All calculations were made according to the manufacturer’s instructions. Four replicates were performed for each group.

## 3 Results and Discussion

### 3.1 Redesign of Preparation Method and Obtained Thermoresponsive Hybrid Nanospheres

We started from a miniemulsion and solvent–evaporation method successfully used to obtain PLGA/PVA nanospheres loaded with NPs.^20,21^ The replacement with PNIPAM of part of the PLGA from the starting organic solution resulted in the necessity of process redesign (see Supporting Information for details). A scheme of the final process, explained through five steps, is shown in Figure 1a. Interestingly, the original tip-ultrasonication step used to form the miniemulsion could be avoided (see Scheme S1), taking advantage of the potential of PNIPAM to create its own miniemulsion above the LCST owing to its coil-to-globule transition (step 2 in Figure 1a). Also, the final water-consuming purification by dialysis (see Scheme S1) could be replaced by a simple process of separation by decantation, after which the aqueous decant (step 4 in Figure 1a) exhibited the best colloidal stability over time.

**Figure 1:**
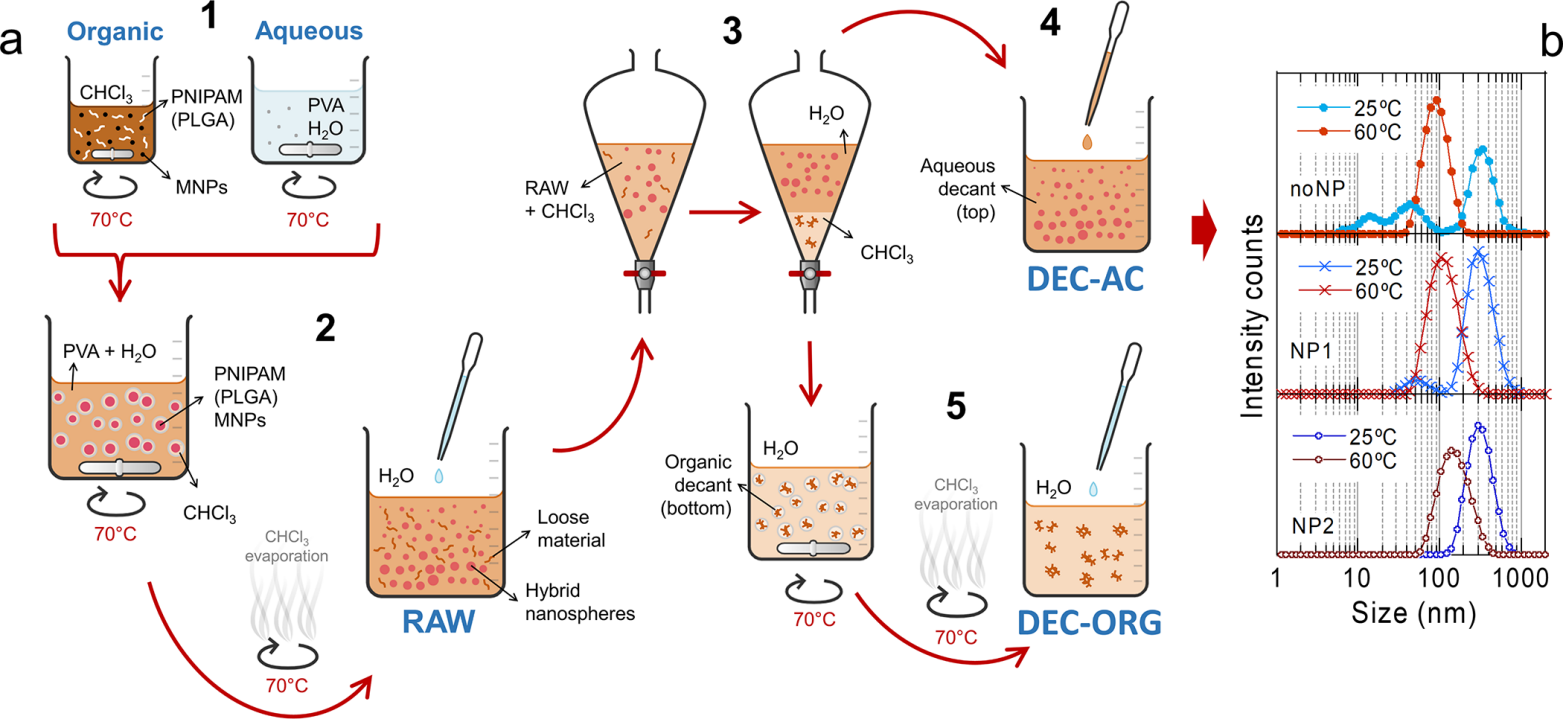
Generic preparation scheme and DLS characterization for uncrosslinked thermoresponsive hybrid nanospheres. (a) Preparation scheme explained through 5 steps. Step 1: starting solutions. The organic solution contained suspended NPs (if any), CHCl_3_ and dissolved PNIPAM (and PLGA). The aqueous solution contained PVA dissolved in distilled water. Once formed by stirring, they were heated on a hot plate at 70 °C. Step 2: miniemulsion evaporation. After, both solutions were mixed heating and stirring, this leading to a miniemulsion. The formation of hybrid nanospheres + loose material occurred upon CHCl_3_ evaporation. Evaporated water during this process was replaced. This sample was named RAW sample. Step 3: separation by decantation. A fraction of the RAW sample was placed into a separating funnel with CHCl_3_, shaken and left to stand until two phases could be clearly distinguished. Step 4: extraction of the aqueous decant (top) with a pipet (named DEC–AC sample). Step 5: extraction of the organic decant (bottom). The decant remaining in the funnel was poured onto hot stirred distilled water and left until complete evaporation of CHCl_3_, after which the evaporated water during the process was replaced (named DEC–ORG sample). (b) Intensity DLS distributions for aqueous decants prepared without NPs (top), with NP1 NPs (middle) and with NP2 NPs (bottom).

Altogether, the method is even more simple and allows to successfully obtain aqueous dispersions of uncrosslinked PNIPAM/PLGA/PVA nanospheres, loaded or not with NPs, and starting directly from PNIPAM, i.e., in a process where polymerization and nanosphere conformation take place separately, in contrast with the extended methods for synthesizing PNIPAM-based nanogels. In addition, these nanospheres, whose nominal PNIPAM mass was only 12% of the total mass, are thermoresponsive. A proof of this can be seen in Figure 1b, which shows the DLS characterization of three aqueous decants, prepared without NPs (top), with NP1 NPs (middle) and with NP2 NPs (bottom). In particular, the intensity distributions are displayed, to emphasize the larger particles corresponding to nanospheres. This supports the formation of relatively homogeneous nanospheres in all cases, whose size clearly decreases above the LCST from ca. 300 nm at 25 °C to ca. 100 nm at 60 °C (well below and above the LCST, respectively). The four next sections are devoted to understanding different aspects of this successful preparation method, by first studying free PVA and PNIPAM in aqueous solutions, and then selected nanosphere suspensions, covering three stages of the process and/or different compositions in terms of types or quantities of polymers and magnetic nanoparticles.

### 3.2 Study of Free PVA and PNIPAM in Aqueous Solution

PVA and PNIPAM are both water-soluble polymers (PNIPAM below the LCST) and thus can appear dissolved in aqueous media after nanosphere preparation. While free PVA is easily removed by dialysis, eliminating free PNIPAM is not straightforward.^18^ To help determining and distinguish the presence of these free polymers in the prepared nanosphere suspensions, PVA and PNIPAM aqueous solutions were first evaluated at concentrations of 7.5 and 5.0 mg/mL, respectively.

Alternate DLS measurements at 25 and 60 °C (below and well above the LCST of PNIPAM) for both aqueous solutions were recorded and averaged (Table 1). Figure 2a collects volume distributions, to show a more balanced landscape between small and large particles. As expected, the behavior of the PVA solution was similar at both temperatures and the distributions show sizes below 20 nm. For PNIPAM, the distribution at 25 °C shows sizes below 50 nm but that at 60 °C displays a high-sized peak (164 nm). Thus, this preliminary analysis indicated that DLS at 25 °C is useful for detecting the presence of dissolved polymers through the presence of peaks at small sizes. In addition, this approach allows us to distinguish between free PVA and free PNIPAM at 60 °C. TEM images of the PNIPAM solution left dry both at room temperature (Figure 2b) and on a hot plate at 70 °C (Figure 2c) are compatible with the DLS results since they show that most particles are smaller than 100 nm at room temperature and vice versa at 70 °C. Agglomerates are also present, some of which could have formed upon drying.

**Table 1:** DLS Intensity, Volume, Number and Zeta Potential Statistics for the PVA and PNIPAM Solutions and 3P Suspensions

			intensity		volume		number		ZP	
sample	*T* (°C)^a^	state	*d*_m_ (nm)^b^	σ (nm)^b^	*d*_m_ (nm)^b^	σ (nm)^b^	*d*_m_ (nm)^b^	σ (nm)^b^	ZP (mV)^c^	σ_ZP_ (mV)^e^
Sol-PVA	25		15	7	9	3	7	1	–6	8
	60		22	13	10	6	4	1	–5	7
Sol-PNIPAM	25		35	14	23	10	14	6	–8	10
	60		224	64	178	43	150	27	–30	11
3P-noNP	25	RAW	715	189	10	3	8	2	–4	5
		DEC–AC	388	137	11	5	6	1	–4	5
		DEC–ORG	530	185	124	64	102	31	–15	7
	60	RAW	160	49	124	32	102	19	–6	5
		DEC–AC	108	41	75	22	59	13	–3	8
		DEC–ORG	118	46	80	30	64	17	–17	8
3P-NP1	25	RAW	309	76	16	7	17	9	–3	4
		DEC–AC	380	155	42	12	33	7	–2	5
		DEC–ORG	256	59	22	3	22	3	–17	10
	60	RAW	157	47	123	31	102	20	–7	6
		DEC–AC	143	68	83	27	62	14	–4	4
		DEC–ORG	116	30	97	23	86	18	–20	10
3P-NP2	25	RAW	1200	553	232	104	171	59	–2	4
		DEC–AC	374	147	251	79	193	46	–2	6
		DEC–ORG	634	454	249	122	151	57	–3	6
	60	RAW	331	93	264	61	225	38	–2	8
		DEC–AC	199	101	109	36	81	17	–5	6
		DEC–ORG	230	112	134	40	104	21	–13	8

a Temperature at which DLS distributions were recorded.

b *d*_m_ and σ are the mean values and standard deviations, respectively, calculated from the parameters *d*_LN_ and σ_LN_ obtained from the lognormal fit of the distributions (see Table S1), considering only the main peak in each case.

c ZP and σ_ZP_ are the mean values and standard deviations, respectively, of the zeta potential measurements.

**Figure 2:**
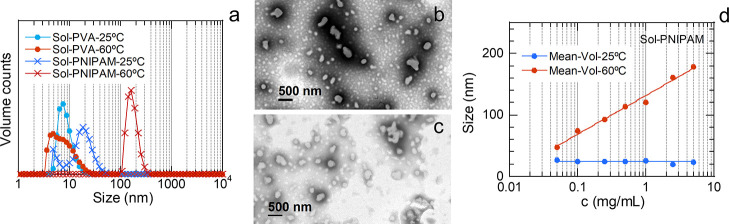
Characterization of PVA and PNIPAM aqueous solutions. The lines in the DLS distributions guide the eyes. (a) DLS volume distributions at 25 and 60 °C. (b,c) TEM micrographs of the PNIPAM solution left dry on carbon-coated Cu grids at room temperature (b) and on a hot plate at 70 °C (c). (d) Mean values of volume distributions of PNIPAM solution at 25 and 60 °C at the studied concentrations. The former was fitted to a constant (24 nm) and the latter to a logarithmic fit, *y* = *a* + b*log­(*x*), where *a* = 132 and *b* = 63.

The observed PNIPAM behavior can be explained based on known dynamics. On the one hand, due to its characteristic coil-to-globule transition, free PNIPAM chains^26^ (peak at 19 nm) can form nanospheres (peak at 164 nm) upon heating above the LCST, and this situation can be reverted upon cooling below the LCST.^27^ On the other hand, the behavior of PNIPAM may vary with solution concentration. For example, free PNIPAM chains can form loose aggregates below the LCST; counterintuitively, this occurs more likely as the concentration of the solution decreases.^28^ Due to this size dependence of PNIPAM on solution concentration, we extended the DLS study of PNIPAM down to a concentration of 0.05 mg/mL (Figures 2d and S3), since free PNIPAM is expected to appear at a lower concentration in nanosphere suspensions than in the starting aqueous solutions. At 25 °C, the small size attributed to free chains remains constant. However, at 60 °C, distributions point to a gradual size reduction of the globules with decreasing concentration following a logarithmic trend. This size reduction makes sense since, given that PNIPAM globules are formed by several chains, reducing solution concentration implies that each globule is formed with fewer chains. These dynamics will be considered when interpreting DLS distributions involving PNIPAM.

The zeta potential data (Table 1) show that PVA is slightly negatively charged in aqueous solution at 25 and 60 °C (−6 and −5 mV, respectively), similar to PNIPAM at 25 °C (−8 mV). These small ZP values predict a poor electrostatic stability of colloidal objects with PVA and PNIPAM on surface. However, PVA redresses this effect by acting as an effective steric stabilizer for PNIPAM in water.^29^ At 60 °C, PNIPAM presents increased electrostatic stability with a ZP of −30 mV. This can be explained by the increase in surface charge density occurring upon coil-to-globule transition, which is simply due to the shrinkage of the polymer chains,^30^ but also owing to an OH^–^ adsorption mechanism.^31^ This particularity will also be exploited to detect the presence of PNIPAM on the surface of the nanospheres.

### 3.3 RAW 3P Suspensions

We then evaluated RAW suspensions of 3P nanospheres (PNIPAM/PLGA/PVA) without and with NPs (3P-noNP-RAW, 3P-NP1-RAW and 3P-NP2-RAW samples). Note that NP2 suspension has a greater concentration than NP1 suspension. Table 1 and Figure 3a present the DLS average results. In addition, Figure S4 accounts for DLS reproducibility and reversible behavior showing several alternate measurements at 25 and 60 °C. At the fabrication temperature (60 °C), the volume distributions (top) display a single peak at a high size, indicating the absence of free material at this temperature. Samples 3P-noNP-RAW and 3P-NP1-RAW exhibit identical distributions, which in turn are similar to those of the PNIPAM solution at a concentration of ca. 1.1 mg/mL (preparation PNIPAM concentration), suggesting that the nanosphere size was fixed by PNIPAM dynamics. The distribution of 3P-NP2-RAW is shifted to much larger values, presumably due to the uptake of a greater number of NPs. Upon cooling to 25 °C, all the distributions become multimodal. Both samples 3P-noNP-RAW and 3P-NP1-RAW present volume peaks at low sizes, which are compatible with the presence of both free PVA and PNIPAM. These results indicate that at 60 °C, when PNIPAM is hydrophobic, all the PVA is located in nanospheres acting as surfactant for PNIPAM and PLGA, while at 25 °C, when PNIPAM is hydrophilic, an excess of PVA and PNIPAM returns to the solution. However, sample 3P-NP2-RAW does not present peaks at low sizes, but it displays two peaks at large sizes, indicative of two populations of nanoobjects. In this case, the greater uptake of hydrophobic NPs results in no excess of free surfactant at 25 °C. Nonetheless, nanospheres were formed within the three samples, as seen in intensity distributions (bottom), which display high-size peaks in all cases. This result leads to the conclusion that the process was successful in obtaining nanospheres, although RAW 3P samples are not ideal due to their multimodality at 25 °C.

**Figure 3:**
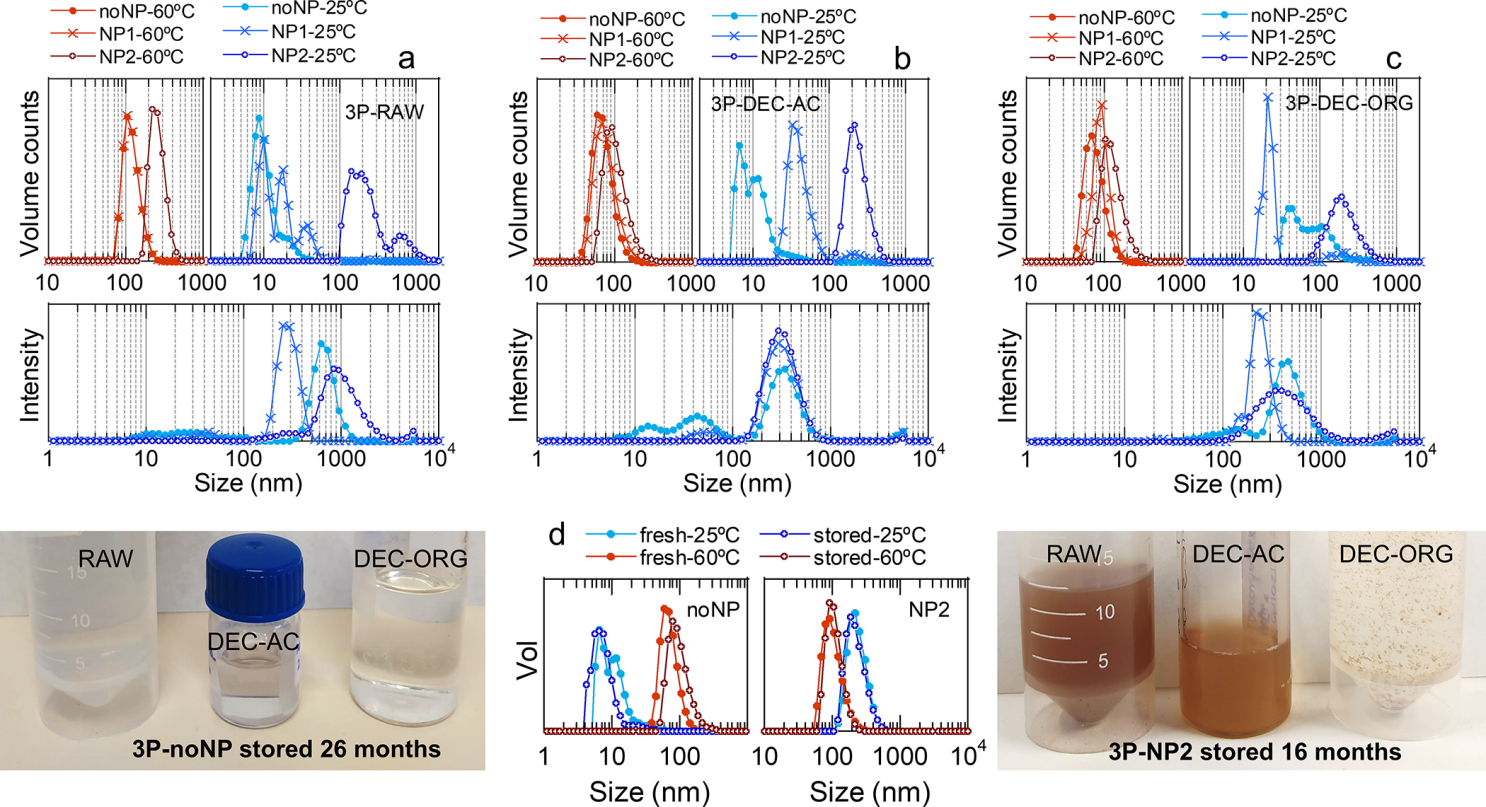
DLS characterization of 3P colloids. (a–c) DLS volume distributions of RAW (a), DEC-AC (b) and DEC-ORG (c) 3P nanospheres at 25 and 60 °C, together with intensity distributions at 25 °C. (d) Images of 3P-noNP (left) and 3P-NP2 (right) suspensions after storage for 26 and 16 months, respectively, together with DLS comparisons (middle) of freshly prepared and stored suspensions. The lines guide the eyes.

ZP values (Table 1) reveal that, similar to PVA, the three samples are slightly negatively charged at both 25 and 60 °C, suggesting that PVA dominates the surface of RAW nanospheres. However, although the RAW samples exhibited good colloidal stability immediately after preparation, they underwent time evolution. Even if most of the volume remains suspended in water, some precipitated material is present, indicative of certain colloidal degradation.

### 3.4 Role of Decantation: DEC–AC and DEC–ORG 3P Nanospheres

#### 3.4.1 Dry Residue and NPs Uptake

After separation by decantation was conducted on the RAW samples, dry residue analysis was performed to quantify the mass distribution between aqueous and organic decants (Table 2). Water retained most of the RAW sample mass (82% on average), while chloroform extracted only 15% on average. Accordingly, DEC–AC samples concentrate the bulk of the preparation output. Additionally, less than 5% of the mass was lost during the decantation process. The mass of magnetic material trapped in both decants was quantified through the combination of elemental analysis and magnetic measurements, as explained in the methods section. We first determined that NP1 and NP2 nanoparticles had a Fe_3_O_4_ weight percent of 70.0% and 34.3%, respectively (the rest was oleylamine and/or oleic acid), and that their *M*_S_ were 63.45 and 51.67 emu/g (grams of Fe_3_O_4_), respectively. These *M*_S_ data, together with the *M*(*H*) curves of the freeze-dried 3P-NP1/NP2-DEC-AC/ORG samples, were used to calculate the magnetic contents; the results are shown in Table 2. In the case of samples 3P-NP1, there is only a small discrepancy (<10%) between the nominal and actual values, but in the case of samples 3P-NP2, which have a much larger NP concentration, this discrepancy increases. This indicates a greater loss of NPs during the processing for these samples. Nonetheless, the NP concentration of 3P-NP2-DEC-AC is five times larger than that of 3P-NP1-DEC-AC. Interestingly, aqueous and organic decants have similar NP concentrations. These results prove the effective integration of NPs in our thermoresponsive nanospheres.

**Table 2:** Evaluation of 3P DEC–AC and DEC–ORG Suspensions

		dry residue evaluation			combined magnetic/elemental analysis		
sample	state	concentration (mg/mL)^a^	% DEC mass^b^	% lost mass^c^	*M*_S_ (emu/g)^d^	c_Fe_3_O_4_,_ _NOM_ (g/g)^e^	c_Fe_3_O_4__ (g/g)^f^
3P-noNP	DEC–AC	9.0	86.4	0.3			
	DEC–ORG	0.8	13.3				
3P-NP1	DEC–AC	7.1	79.3	3.8	63.45	1.18 × 10^–3^	1.09 × 10^–3^
	DEC–ORG	0.9	17.3				1.08 × 10^–3^
3P-NP2	DEC–AC	5.1	81.1	4.3	51.67	7.66 × 10^–3^	5.73 × 10^–3^
	DEC–ORG	0.8	14.6				4.80 × 10^–3^

a Mass concentration of the dry residue.

b Mass percentage of RAW samples found in DEC suspensions.

c Mass percentage lost due to decantation.

d Saturation magnetization (*M*_S_) of NP1 and NP2 nanoparticles.

e Nominal concentration of Fe_3_O_4_ in nanospheres according to the quantities used in the fabrication of RAW samples.

f Measured concentration of Fe_3_O_4_ in nanospheres.

#### 3.4.2 DLS and Zeta Potential

DLS results (Table 1 and Figure 3b,c; see Figure S4 for reproducibility and reversible behavior) of the three DEC-AC and three DEC–ORG samples show distinct distributions, supporting the utility of the separation process. Similar to RAW samples, all DEC–AC samples display a single volume (top) peak at 60 °C, whose size increases as the NP concentration increases, that is, following the sequence noNP-NP1-NP2. But upon cooling to 25 °C, only noNP and NP1 distributions become multimodal. In sample 3P-noNP-DEC-AC, the main volume peak at 25 °C (*c.a.* 9 nm) corresponds to free PVA. Sample 3P-NP1-DEC-AC lacks the PVA volume peak, instead a peak at ca. 37 nm becomes dominant. Since no free PNIPAM is expected in the DEC–AC samples, this peak could be ascribed to small nanoobjects including both PVA and PNIPAM. Sample 3P-NP2-DEC-AC displays only one volume peak at large size (221 nm), proving the efficacy of the method for producing monomodal 3P nanospheres loaded with nanoparticles that are free of precursors solubilized in water. Anyhow, NPs clearly modulate the polymer mass contribution to nanospheres, since size monomodal nanospheres (with no free material) were achieved only with the highest NP uptake, suggesting different optimal preparation quantities for few or no NPs. Also, the size of 3P-NP2-DEC-AC nanospheres at 60 °C was much smaller than that at 25 °C, suggesting that the thermal cycling process was similar to that of PNIPAM-based crosslinked hydrogels (i.e., the size increased upon cooling). In particular, the volume mean size of these nanospheres decreased to 43% of the initial size upon heating. Moreover, the three intensity (bottom) distributions show a very similar peak at large size, which is indicative of the presence of nanospheres of the same size in all the DEC–AC samples, with equal thermal cycling behavior. In addition, the ZP values indicate that DEC–AC samples are slightly negatively charged at both 25 and 60 °C, similar to both the RAW samples and the PVA solution, indicating predominant PVA coverage.

Similar to RAW and DEC–AC, all DEC–ORG samples display a single volume (top) peak at 60 °C, whose size increases as the NP concentration increases. Also, none of the volume distributions at 25 °C showed free PVA. However, although volume sizes at 60 °C would be compatible with those of free PNIPAM at several concentrations (Figure 2d), volume sizes at 25 °C are not fully compatible with free PNIPAM, thus indicating the presence of nanoobjects also in the organic decant. In particular, sample 3P-NP2-DEC-ORG shows single large-sized peaks at both 25 and 60 °C, which are shifted to lower and greater sizes, respectively, than those of 3P-NP2-DEC–AC. These nanospheres are reduced to 54% of their initial mean volume size upon heating, thus displaying a smaller size reduction than 3P-NP2-DEC–AC. In contrast to those of the RAW and DEC–AC samples, the ZP values of samples 3P-noNP-DEC–ORG and 3P-NP1-DEC–ORG were already −15 and −17 mV, respectively, at 25 °C. This fact indicates that these nanospheres contain packed PNIPAM on the surface since free PNIPAM would appear only slightly negatively charged at 25 °C. This is probably the reason why these nanospheres are dragged downward by chloroform during the decantation process. Upon heating to 60 °C, the ZP values became slightly more negative (−17 and −20 mV). Instead, sample 3P-NP2-DEC-ORG displays a ZP of −3 mV at 25 °C but more negative values at 60 °C (−13 mV). This difference surely arises from the greater NP content in this sample and is still compatible with the presence of PNIPAM chains on the surface of the nanospheres, which in turn justifies their preference for chloroform.

#### 3.4.3 Colloidal Stability

Although DEC–AC and DEC–ORG samples, like RAW samples, exhibited good colloidal stability immediately after preparation, some of the samples underwent changes over time. Figure 3d displays images of the states of samples noNP and NP2 after storage periods of 26 and 16 months, respectively, after a short vortex agitation. Some precipitated material is perceived at the bottom of the RAW samples, while the DEC–AC samples are fully suspended. In particular, sample 3P-NP2-DEC–AC was a lighter orange color than was 3P-NP2-RAW. A comparison of the DLS volume distributions before and after storage revealed that 3P-noNP-DEC–AC had a certain increase in nanosphere size at 60 °C, but the nanosphere size of 3P-NP2-DEC–AC did not vary. Instead, DEC–ORG samples are full of fragments that readily decant, indicating severe colloidal degradation. In summary, the applied decantation process has succeeded in separating water-unstable materials. DEC–AC samples, especially 3P-NP2-DEC–AC, which displayed no loose material according to DLS analysis, were stable over time. As revealed by the ZP, this stability is achieved by the PVA on surface.

#### 3.4.4 TEM and Cryo-TEM

Decanted 3P-noNP (Figure S5) and 3P-NP1 samples could be observed by TEM simply by dropping them onto carbon-coated Cu grids and allowing them to dry, since the presence of free polymer chains led to the formation of a film upon drop casting, which was evident to the eye and protected the nanospheres from drying or shrinking. Figure 4a–f correspond to sample 3P-NP1-DEC–AC. Figure 4a,c show TEM images of sample 3P-NP1-DEC–AC prepared at room temperature and at different magnifications. Nanospheres of ca. two hundred nanometers in size are clearly appreciable owing to the negative staining. The nanospheres displayed an inner white contrast, an outer gray contrast and very few black points corresponding to the NPs. According to previous TEM studies on PVA/PLGA nanospheres,^21^ one can assume that the inner white contrast corresponds to PLGA and that the gray contrast corresponds to the hydrophilic polymers PVA and/or PNIPAM. In addition, Figure 4b,d show TEM images of the same sample dried on a hot plate at 70 °C. A comparison of the images a and b (and c and d) reveals the occurrence of the size reduction displayed by DLS, except for a few nanospheres that failed to undergo the transition. A closer look (Figure 4d) indicates that these small nanospheres have the same contrast distribution as the larger ones. In addition, a higher TEM magnification of this sample dried on a hot plate (Figure 4e) revealed the size reduction mechanism followed by these nanospheres. Upon heating, small nanospheres start to protrude from large ones, so that small nanospheres are the product of the disintegration of large nanospheres. Notably, the absence of PNIPAM crosslinking implies the presence of a size reduction mechanism distinct from the shrinking/swelling of PNIPAM microgels,^32^ namely, disintegrating/rebuilding, and this process is reversible according to DLS (Figure S4). Finally, the size distributions obtained from the histograms of these images were compared (Figure 4f) to the number distributions calculated from DLS (for the number calculation at 25 °C, only the high-size peak was used). A great similarity was found, indicating that the nanospheres retained their original dimensions upon grid preparation and that the images are representative of the nanospheres in suspension.

**Figure 4:**
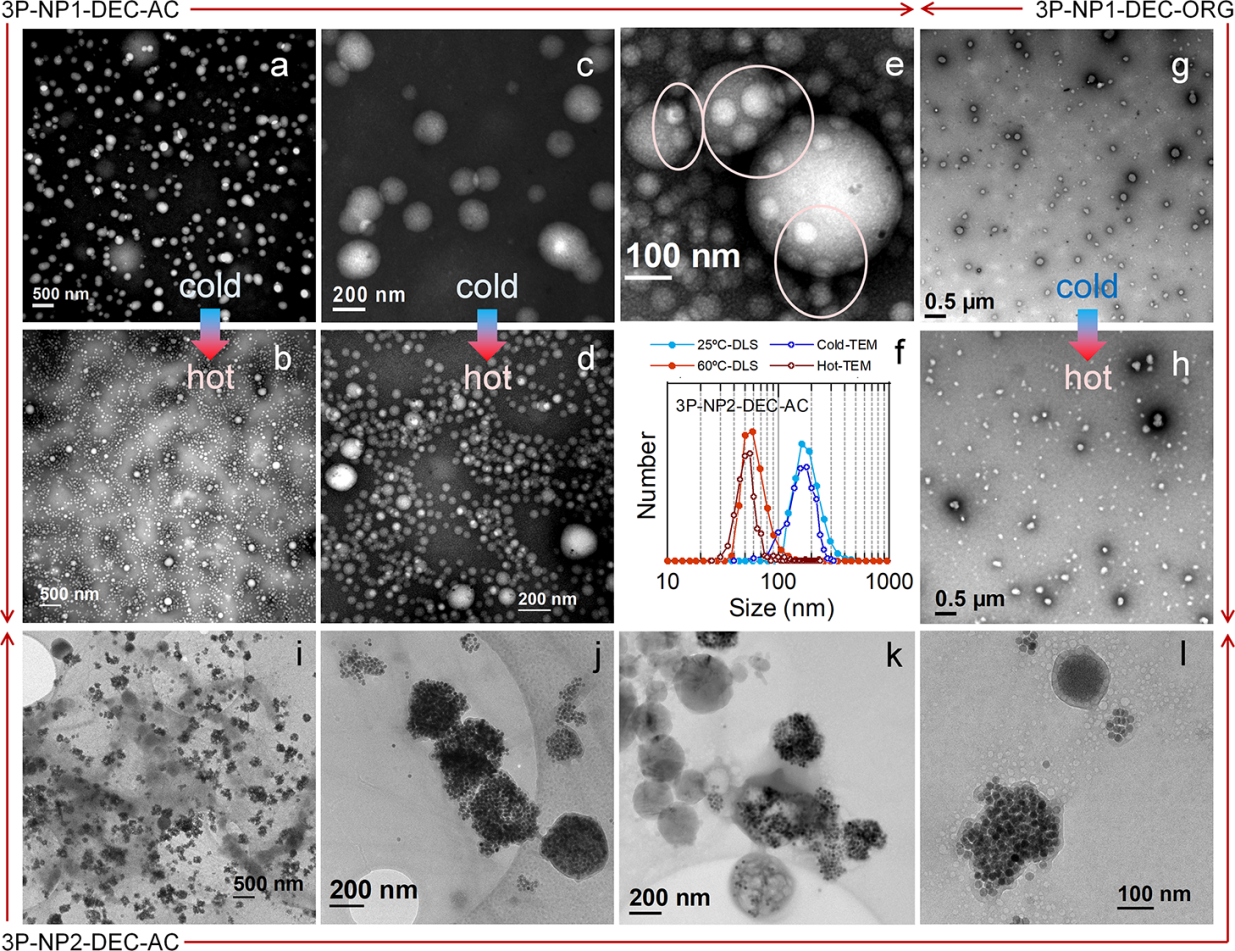
TEM and cryo-TEM analysis of 3P nanospheres. (a–e) Negatively stained TEM micrographs of 3P-NP1-DEC–AC left dry on carbon-coated Cu grids at room temperature (cold-TEM: a,c) and on a hot plate at 70 °C (hot-TEM: b,d). Note that images (a,b) are at the same magnification, as are images (c,d). (e) Detail of (d) highlighting small nanospheres protruding from large ones (pink circles). (f) DLS number distributions of 3P-NP1-DEC–AC at 25 and 60 °C, together with TEM distributions obtained from cold-TEM and hot-TEM micrographs. (g,h): TEM micrographs of 3P-NP1-DEC–ORG left dry on carbon-coated Cu grids at room temperature (g) and on a hot plate at 70 °C (h). (i–l) Cryo-TEM micrographs of 3P-NP2-DEC–AC displaying different degradation stages, in which core–shell structures and nanoparticles can be observed.

Figure 4g,h show TEM images of sample 3P-NP1-DEC–ORG prepared at room temperature and dried on a hot plate. A size reduction is also visible, although the nanospheres appear deformed and have similar morphologies to those of free PNIPAM agglomerates.

Decanted 3P-NP2 samples were not stable upon drop casting on TEM grids, and many nanosphere shells (polymers + NPs) were semidetached from the cores (white contrast) in the captured micrographs (not shown). Cryo-TEM succeeded better than drop casting (Figure 4i–l). However, given that the nanosphere diameter was relatively large compared to the thickness of the ice film expected to form on the grids,^33^ most of the nanospheres were deformed or broken, and no size distribution histograms were obtained. Even so, it was possible to observe some preserved nanospheres with a polymeric shell and some nanospheres that still displayed a core–shell distribution, with many NPs attached at the core–shell interface, confirming high NP uptake.

#### 3.4.5 Thermal Analysis

Thermal analysis was conducted on DEC–AC and DEC–ORG 3P nanospheres aiming to get more information about the polymeric part of the nanospheres. DSC was first performed on the raw polymers to determine their glass transition temperature (*T*_g_) and heat capacity (*C*_p_) (Figure 5a). PLGA, PVA and PNIPAM had well-distinguishable *T*_g_ values (34.2, 67.3, and 137.5 °C, respectively). Additionally, PVA exhibited the highest *C*_p_, PLGA lay in the middle and PNIPAM followed far behind. Second, several PVA/PNIPAM mixtures were prepared and subjected to DSC. Figure 5a shows that a PVA/PNIPAM powder mixture of ca. 80/20 in mass had both *T*_g_ and *C*_p_ values in accordance with the rule of mixtures. The 50/50 dried aqueous solutions, both prepared at room temperature (cold solution) and heated on a hot plate at 70 °C, also displayed *C*_p_ values following the rule of mixtures. However, their *T*_g_ values varied. In particular, the *T*_g_ initially corresponding to PVA slightly increased to ca. 70 °C, and that of PNIPAM decreased to ca. 125 °C, although the cold solution still provided part of the original *T*_g_ of PNIPAM. These shifts point to a partial miscibility between PVA and PNIPAM, giving rise to polymer blends. It is known that the analysis of these *T*_g_ shifts is helpful in the study of polymer blends, although interpretation is not straightforward. The *T*_g_ of the blend depends on the relative weight of each polymer, but given that there are many other factors affecting its properties, the *T*_g_-weight relationship barely fulfills the rule of mixtures. Several models for *T*_g_ prediction have been developed^34,35^ but must be applied with caution. Accordingly, *T*_g_ shifts were discussed only qualitatively.

**Figure 5:**
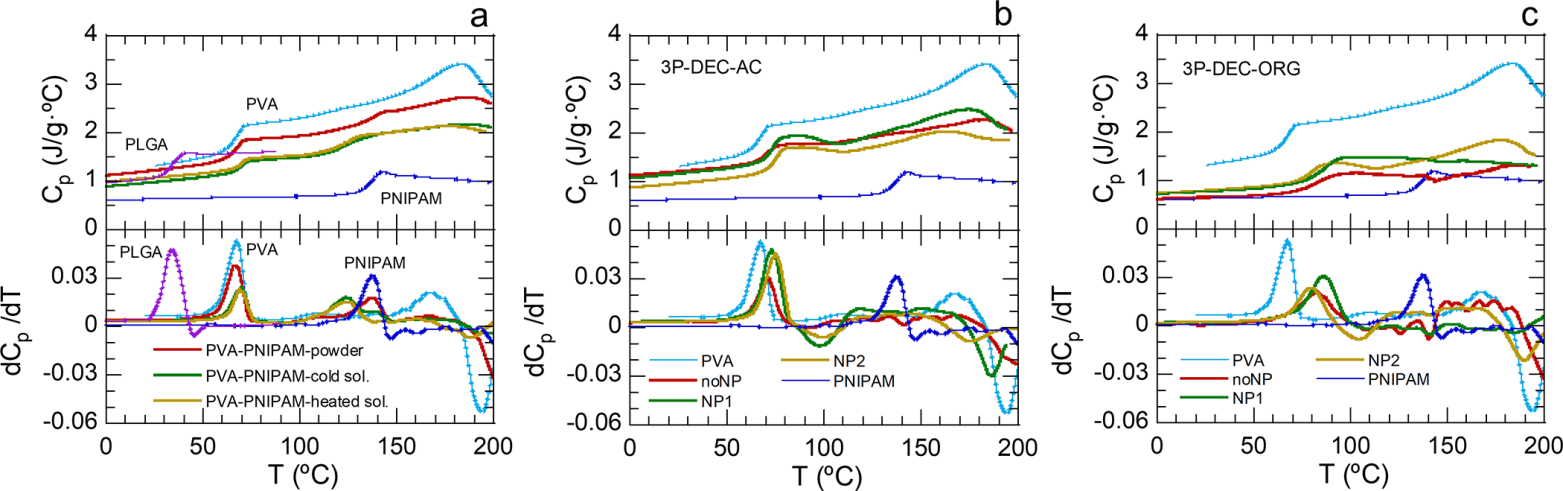
Thermal analysis by DSC of starting polymers and 3P nanospheres. Upper plots: heat capacity evolution with temperature. Lower plots: numerical derivative of the heat capacity with respect to temperature. From these plots, the glass transition temperature was chosen at the slope inflection point of the glass transition step (derivative maxima). (a) Starting polymers (PVA, PLGA and PNIPAM) of 3P samples, together with PVA/PNIPAM mixtures in powder (ca. 80/20), aqueous solution at room temperature (50/50) and aqueous solution heated on a hot plate at 70 °C (50/50). (b) 3P-DEC–AC samples together with PVA and PNIPAM. (c) 3P-DEC–ORG samples with PVA and PNIPAM.

The six freeze-dried 3P-decanted samples were analyzed. Neither of them showed the *T*_g_ of PLGA, which was expected, given the low mass percentage of this polymer in all the samples. Figure 5b displays the results for the DEC–AC samples. All the *C*_p_ values were closer to those of PVA than to those of PNIPAM, which is in accordance with the nominal nanosphere composition, with a majority of PVA. Sample 3P-noNP-DEC–AC displayed two *T*_g_ values, one initially corresponding to PVA, which shifted upward to 70.9 °C (similar to that of PVA/PNIPAM solutions), and a small peak at 133.1 °C, assigned to the *T*_g_ of PNIPAM but slightly shifted downward. Samples 3P-NP1-DEC–AC and 3P-NP2-DEC–AC, however, did not present a *T*_g_ for PNIPAM; rather, only the *T*_g_ for PVA shifted to 73.2 and 74.9 °C, respectively. This result indicates the presence of a unique miscible blend containing an increasing amount of PNIPAM. In addition, Figure 5c displays the results for the DEC–ORG samples. These three samples had *C*_p_ values closer to those of PNIPAM, indicating the presence of a majority of this polymer, which could explain their preference for chloroform in the decantation process. As its DEC–AC analogous sample, 3P-noNP-DEC–ORG displayed two *T*_g_: one corresponding to that of PVA but shifted to 83.3 °C, which was higher than that of 3P-noNP-DEC–AC, in agreement with a higher PNIPAM content; and the other at 134 °C, which was very similar to that of 3P-noNP-DEC–AC. Again, as their DEC–AC analogous samples, 3P-NP1-DEC–ORG and 3P-NP2-DEC–ORG did not present the *T*_g_ of PNIPAM. Instead, they showed a *T*_g_ corresponding to PVA but shifted to 86.1 and 79.6 °C, respectively, in accordance with a higher PNIPAM content than DEC–AC samples. In summary, DSC demonstrated the formation of different polymer nanoblends within the nanospheres. Additionally, the decantation process is able to effectively separate the different blends between the aqueous and organic fractions, especially in the samples with magnetic nanoparticles, in which a unique *T*_g_ is ultimately obtained within each fraction. In addition, according to both the *C*_p_ and *T*_g_ values, the DEC–AC fraction contained more PVA than the DEC–ORG fraction; hence, the DEC–AC fraction was nearer to the nominal composition. These results are consistent with the observed good/poor colloidal stability of the DEC–AC/DEC–ORG samples, respectively. In DEC–AC samples, the PVA-rich blends formed ensure that enough PVA is effectively integrated on the nanospheres so as to achieve their steric stabilization.

### 3.5 Role of PLGA: 2P Nanospheres

3P samples were fabricated using a mass ratio of 85/15 of PNIPAM/PLGA in the organic solution. We prepared analogous samples within the PVA/PNIPAM system (2P-noNP, 2P-NP1 and 2P-NP2), i.e., substituting PLGA with PNIPAM, to evaluate the role of PLGA in 3P samples. First, 2P nanospheres exhibited an important time evolution from the moment of preparation/decantation, where DLS showed very wide distributions, to approximately 48 h after preparation. Table 3 collects the DLS results after this delay time. The distributions of 2P-noNP-DEC–AC and 2P-NP1-DEC–AC (Figure S6) resembled their 3P counterparts, but those of the 2P samples were notably wider and shifted to greater sizes. Then, PLGA seems to have an important role in nanosphere size and dispersity. In addition, Figure 6 displays the comparison between 2P-NP2-DEC–AC (in the middle) and its 3P counterpart. A significant difference was found, since the 2P distribution at 25 °C was multimodal, contrarily to that of 3P, and that at 60 °C was shifted to much lower values. This outcome led us to investigate whether a more adequate PVA/PNIPAM ratio could produce monomodal distributions for 2P-NP2. For this purpose, assuming the nominal sample 2P-NP2 to be 100% PVA, we prepared four more samples 2P-NP2 (25% PVA, 50% PVA, 200% PVA and 400% PVA). The DLS volume results for the DEC–AC samples are shown in Table 3 and depicted in Figure 6 (see also Figure S6).

**Table 3:** DLS Intensity, Volume, Number and Zeta Potential Statistics for 2P DEC–AC Suspensions

			intensity		volume		number		ZP	
sample	% PVA	*T* (°C)^a^	*d*_m_ (nm)^b^	σ (nm)^b^	*d*_m_ (nm)^b^	σ (nm)^b^	*d*_m_ (nm)^b^	σ (nm)^b^	ZP (mV)^c^	σ_ZP_ (mV)^c^
2P-noNP	100	25	352	202	9	2	8	1	–2	5
		60	158	73	94	32	70	17	–3	10
2P-NP1	100	25	723	679	12	6	8	2	–1	4
		60	172	78	105	34	79	18	–1	6
2P-NP2	25	25	485	440	143	72	78	30	–10	11
		60	221	107	127	41	96	22	–14	9
	50	25	242	132	128	47	96	20	–2	6
		60	173	69	116	34	91	19	–8	8
	100	25	709	455	131	84	90	43	–1	5
		60	500	392	61	19	49	12	–7	10
	200	25	336	169	185	66	134	32	–1	4
		60	174	76	107	34	82	18	–3	5
	400	25	433	253	208	84	140	41	–3	6
		60	284	167	134	57	88	26	–5	6

a Temperature at which DLS distributions were recorded.

b *d*_m_ and σ are the mean values and standard deviations, respectively, calculated from the parameters *d*_LN_ and σ_LN_ obtained from the lognormal fit of the distributions (see Table S2), considering only the main peak in each case.

c *ZP* and σ_ZP_ are the mean values and standard deviations, respectively, of the zeta potential measurements.

**Figure 6:**
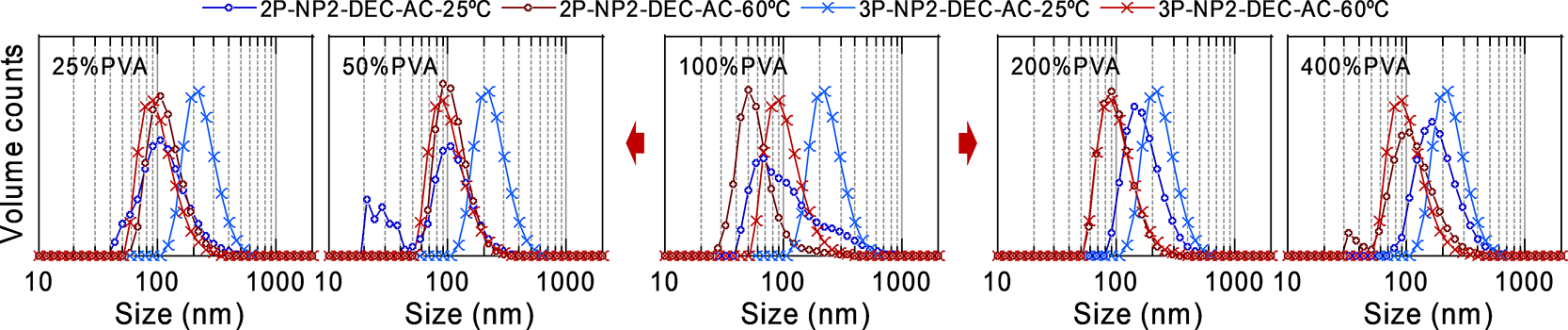
DLS volume distributions of the 2P-NP2-DEC–AC samples obtained by ranging the mass percentage of PVA between 25 and 400%, with 100% being the nominal value, and comparison with 3P (100%) analogue. The lines guide the eyes.

The first notable feature is that, except for 2P-NP2 (100% PVA) and the small peak of 2P-NP2 (50% PVA), the distributions are monomodal. On the one hand, one can observe that the 25 and 50% PVA samples undergo essentially no size reduction between 25 and 60 °C. This result is not obvious given that these samples have the highest PNIPAM contents. On the other hand, the 200 and 400% PVA samples are thermoresponsive, and their sizes decreased to 57 and 64%, respectively, less than that of the 3P-NP2-DEC–AC sample (42%). Distributions at 60 °C (except that of 100% PVA) show similar percentage standard deviations and only slightly different sizes, which in turn coincide with those of 3P-NP2-DEC–AC. In conclusion, PVA contents lower/higher than 100% hinder/allow size changes in 2P nanospheres. Additionally, when comparing 2P and 3P nanospheres, the role of PLGA seems 2-fold: (i) narrowing of the size distribution at 25 °C in nanospheres with few or no NPs and (ii) reducing the absolute % of PVA necessary to obtain a significant increase in size upon cooling in highly NP-loaded nanospheres. Eventually, the ZP of the 2P-NP2-DEC-AC samples evolves from more negative mean values and wider standard deviations at 25% PVA to values very similar to those of 3P-NP2-DEC-AC at 200 and 400% PVA. This highlights that a higher PVA content is required in 2P samples to achieve characteristics comparable to those of 3P samples.

### 3.6 Aptitude for Biomedical Applications

As mentioned before, the previous sections address different aspects related to the obtaining of uncrosslinked thermoresponsive hybrid magnetic nanospheres through the study of diverse selected samples. Nevertheless, the most adequate nanospheres are those collected in aqueous decants. In particular, the sample that fulfills best the pursued characteristics is 3P-NP2-DEC–AC, since it presents no small objects or loose materials, have nanospheres of 193 ± 46 nm in size that displays important size reduction upon heating across the LCST, shows excellent colloidal stability and exhibit a remarkable uptake of magnetic nanoparticles. For these reasons, we performed on this sample the evaluation of the particular biomedical functionalities of the nanospheres.

#### 3.6.1 Heating Ability

First, with a view toward applications in hyperthermia or heat-assisted drug release, the heating ability was determined in the 10–60 °C temperature range. Figure 7a,b display the calculated heating ability (SAR) in W/g (grams of Fe_3_O_4_) of 3P-NP2-DEC–AC nanospheres in function of the ac magnetic field and frequency, respectively. Also, Figure S2 shows representative heating steps and the SAR of NP2 (just nanoparticles). It was found that the SAR­(*T*) trends of both, nanoparticles and nanospheres, are quite similar except for the absolute values. SAR­(*H*_0_) data were extracted at several temperatures (10, 20, 30, 40, and 50 °C) and are plotted in Figure 7c. Neglecting temperature variation, the data were fitted well to quadratic functions (according to Rosensweig’s model^36^), revealing that the freeze-dried 3P-NP2-DEC-AC nanospheres outperform the dry NP2 nanoparticles by a factor of 1.5. This improvement in SAR, previously observed for non thermoresponsive PVA/PLGA nanospheres,^21^ is related to the particular arrangement of NP2 nanoparticles on the nanosphere surface, which differs from that of dry NPs. However, the TEM images of the 3P-NP2-DEC–AC nanospheres did not reveal the exact surface arrangement, which is an issue that needs to be addressed in the future. In addition, Figure 7d reflects the linear dependence of SAR on *f*.

**Figure 7:**
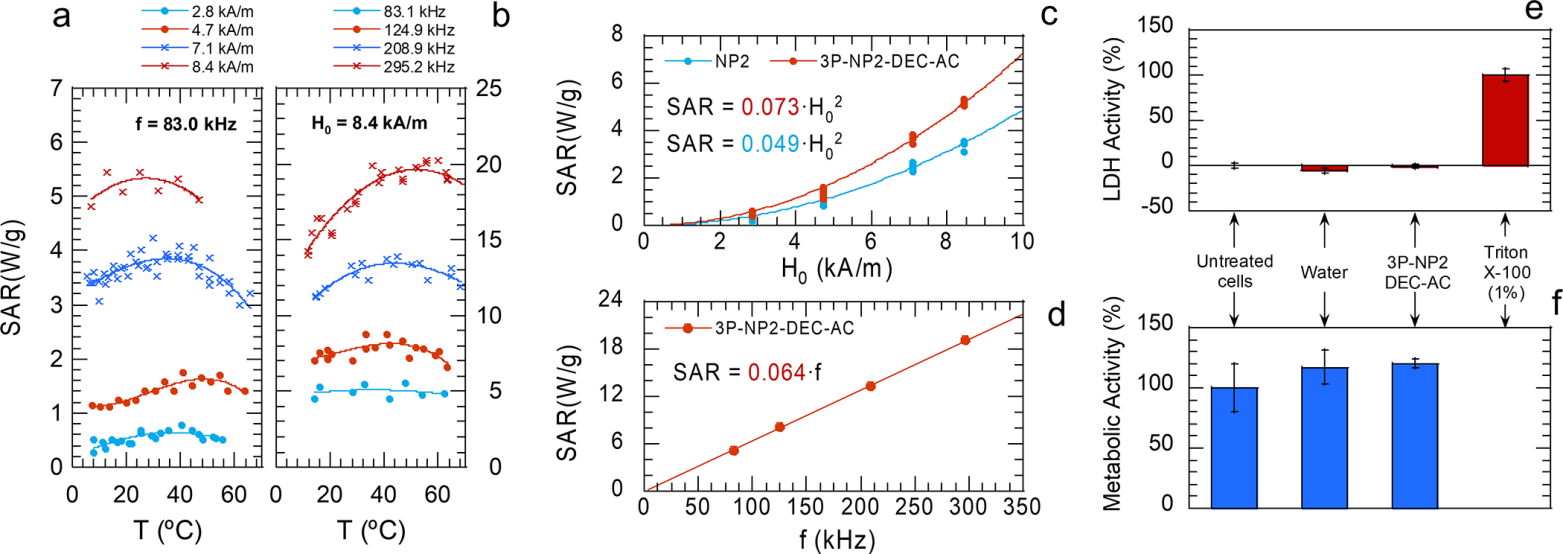
(a–d) Heating ability of 3P-NP2-DEC-AC nanospheres. SAR­(*T*) trends of freeze-dried 3P-NP2-DEC–AC nanospheres, measured for several *H*_0_ (a) and *f* (b) values, and fitted to polynomial functions to guide the eyes. From them, SAR­(*H*_0_) (c) and SAR­(*f*) (d) trends were obtained by extracting isothermal data. The former trend was plotted together with that of NP2 dried nanoparticles, for comparison. SAR­(*H*_0_) fitted well to square power functions and SAR­(*f*) to a linear function, according to Rosensweig’s model. (e) Results of the LDH in vitro assay, in which the percentages are relative to the control (Triton X-100 1%). (f) Results of the resazurin in vitro assay, in which the percentages are relative to the control (untreated cells). The data are presented as the mean ± standard deviation.

With respect to the absolute SAR values and for comparison with those of other nano-objects, it is useful to convert the SAR into the intrinsic loss parameter^37^ (ILP, expressed in nH·m^2^/kg_Fe_). The ILP is a heating ability indicator that aims to eliminate the influence of using more or less powerful applied magnetic fields in SAR determination. According to Rosensweig’s model, the SAR has a linear dependence on the frequency and a quadratic dependence on the amplitude, and this is the basis of the ILP calculation. Although Rosensweig’s model is theoretically valid only for low field amplitudes and because this low limit depends on the characteristics of the material under evaluation, the use of ILP is still better than raw SAR for comparisons even with high-amplitude magnetic fields. In the case of our nanospheres, Figure 7c,d support the use of ILP (= 0.88 nH·m^2^/kg_NP_ for nanospheres). Assuming the safety limits for ac field application established in the so-called Hergt-Dutz criterion^38^ (*H*_0_·*f* ≤ 5 × 10^9^ A·(m·s)^−1^), the SAR values of our nanospheres can largely be increased using higher *H*_0_ and *f* values so that enough heat is generated for biomedical applications. To illustrate this, we first compared our ILP value with that of nanoobjects reported to cause hyperthermic local cell death with a weak global temperature increase.^39^ In particular, the authors found a cell death of 60% with a SAR of 150 W/g_NP_ using 100 kHz and 48 kA/m (≅ limit of the Hergt-Dutz criterion), that is, with a ILP of 0.65 nH·m^2^/kg_NP_, lower than that of our freeze-dried 3P-NP2-DEC–AC nanospheres. Second, we analyzed the possibility of achieving a global temperature increase from 37 to 43 °C with our nanospheres, relying on SAR estimations reported for an idealized tumor phantom containing an intratumor NP concentration of 1 mg/mL.^40^ A SAR value of 90 W/g_NP_ is required that, using 100 kHz and our ILP value, is achieved applying 32 kA/m. Limitation in this case lies on the required intratumor nanosphere concentration: 1 mg/mL of NPs implies 0.14 mL/mL nanospheres, that can be reduced by half (7% of tumor volume) using 100 kHz and 48 kA/m. This is challenging to achieve, assuming that this kind of nanoobjects is collected in cell lysosomes, and lysosomes constitute 1–15% of mammalian cell volume. However, as we proved that our nanospheres can uptake different types of NPs, SAR could be further increased by using more performant NPs (e.g., nanocubes or nanoflowers^41,42^ with ILP values of 8.3–8.6 nH·m^2^/kg_Fe_). In summary, the heating ability of the dried 3P-NP2-DEC–AC nanospheres is good, but there is still room for improvement as regards magnetic NPs.

#### 3.6.2 In Vitro Assays

3P-NP2-DEC–AC nanospheres are expected to be suitable for biomedical applications since magnetite, PVA, PLGA and PNIPAM are known biocompatible materials. However, even though the experimental conditions used ensure full elimination of residual chloroform, considering its thermophysical properties, this solvent is cytotoxic at trace levels. Hence, a primary evaluation of cytotoxicity was performed using the lactate dehydrogenase (LDH) assay, which is indicative of cell death in culture media, and the resazurin assay, which is indicative of metabolic activity in culture media. Figure 7e shows the results of the LDH assay, which indicated that neither the cells treated with water nor those treated with 3P-NP2-DEC–AC nanospheres significantly differed from the untreated cells (negative control), while all the cells treated with Triton X-100 died (positive control). These findings confirmed that 3P-NP2-DEC–AC nanospheres are noncytotoxic at a concentration of 0.5 mg/mL after 24 h of exposure. The cell morphology after treatment (Figure S7) also supported these results. In addition, Figure 7f displays the results of the resazurin assay. The metabolic activity of cells treated with water or 3P-NP2-DEC–AC nanospheres was similar to or slightly greater than that of untreated cells (positive control). As expected, the cells lysed with Triton X-100 exhibited 0% metabolic activity. In summary, the 3P-NP2-DEC–AC nanospheres exhibited metabolic activity above 70%, which is the threshold set by the ISO EN 10993:5 standard. Accordingly, this absence of cytotoxicity confirms indirectly that residual chloroform, if any, is under toxicity levels.

### 3.7 Aptitude as Heating Rate Nanosensors

In the previous sections, we assessed the reproducible size increase/reduction of 3P nanospheres upon direct cooling/heating cycles between 25 and 60 °C. However, when performing stepped heating ramps to locate the position of the LCST, i.e., at conducting intermediate measurements between 25 and 60 °C at fixed temperatures, namely, at 30–31–32–33–34–35–36–38–40 °C, changes were observed. Figure 8 (and Figure S8) shows the DLS results for these stepped heating ramps, together with those previously obtained for direct heating from 25 to 60 °C. In sample 3P-noNP-DEC–AC (Figure 8a), whose small-sized main volume peak was assigned to free PVA, 34 °C seems to be the onset of size change along this heating ramp. From 35 to 60 °C (the average is shown), all the distributions are narrower and already identical. However, the final size after the stepped heating ramp stays considerably larger than that obtained through direct heating between 25 and 60 °C. The results for sample 3P-NP2-DEC–AC (Figure 8b) are essentially similar. The main difference is that, since this sample does not present loose material, the observed changes in the volume distributions reflect better the evolution undergone by the nanospheres across LCST. These size change dynamics are fully reversible since direct and stepped heating ramps were alternatively used to derive reproducible results. Accordingly, the dynamics do not seem to depend on the heating history but rather on the applied heating ramp. Similar stepped heating ramps were applied to the PNIPAM aqueous solution (Figure 8c) and the same qualitative behavior was observed. Accordingly, 3P DEC–AC nanospheres inherited the dynamics from free PNIPAM, although with a modulated final size reduction.

**Figure 8:**
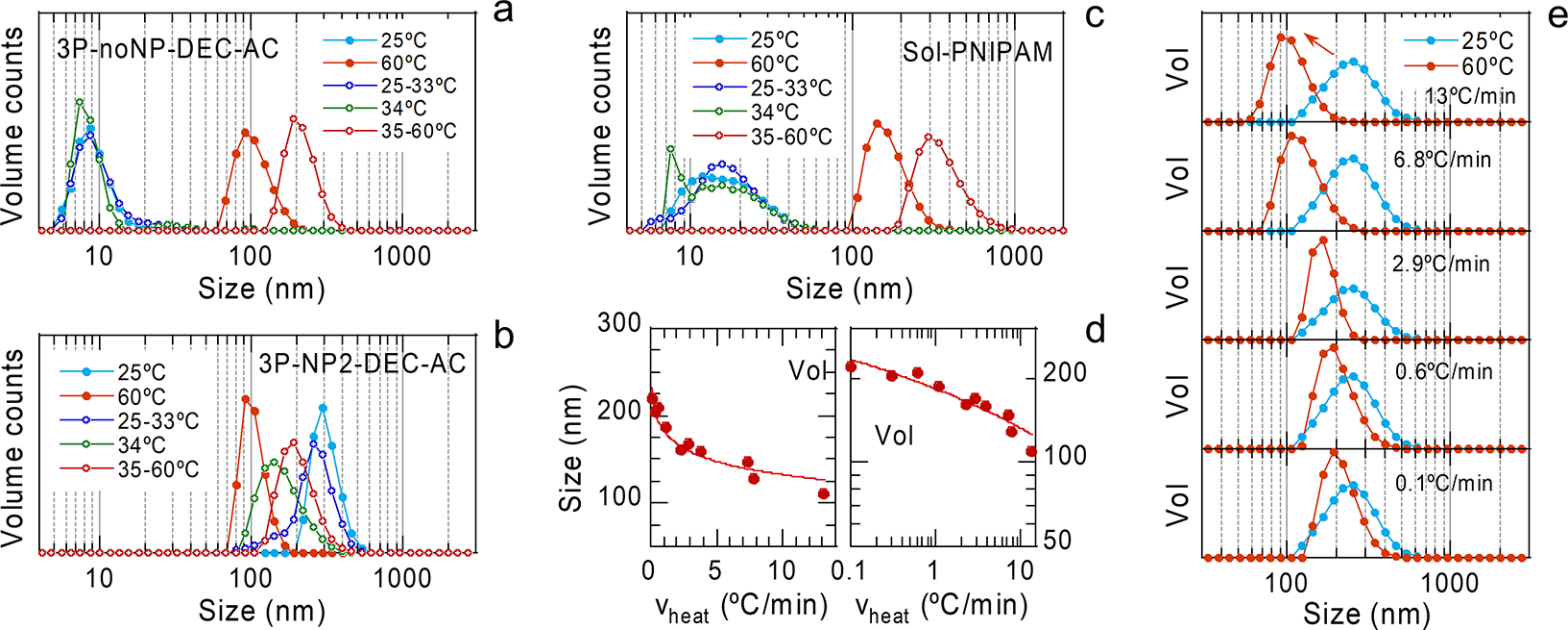
(a–c) DLS volume distributions of 3P-noNP-DEC–AC (a), 3P-NP2-DEC–AC (b) and PNIPAM solution (c) after direct heating between 25 and 60 °C (full symbols) and stepped heating ramps following the sequence 25–30–31–32–33–34–35–36–38–40–60 °C (open symbols). An average is shown for the measurements between 25 and 33 °C and between 35 and 60 °C since they were identical within each interval. (d,e): DLS volume results of 3P-NP2-DEC–AC nanospheres at 25 °C and after heating to 60 °C with the help of a bath with programmable heating ramps ranging between 6.8 and 0.1 °C/min (a 13 °C/min rate is achieved only by DLS). (d) mean size variations at 60 °C, calculated by lognormal fitting volume distributions, as a function of the heating rate (*v*_heat_). Both linear (left) and log–log (right) plots are shown. Lines are fits to logarithmic trends, *y* = *a* – b*log­(*x*), where *a* = 182.55 and *b* = 50.31. (e) Change in volume distributions at selected heating rates, with lines to guide the eyes.

Previous related work was performed on cylindrical PNIPAM gels,^43^ studying their shrinking/swelling behavior from a fixed initial temperature (20 °C) to different final temperatures (30–55 °C), with ca. 1 min of heating time. This led to conclude that the fact of heating to different final temperatures affects PNIPAM kinetics, in the sense that the shrinkage rate (and not the final size, which was similar for all final temperatures after 4000 min) decreased by more than 100 times for certain final temperatures (40 and 45 °C). To evaluate whether the 3P-NP2-DEC–AC nanospheres could undergo a similar decrease in shrinkage rate, a further DLS experiment was conducted in which, after applying a direct ramp from 25 to 40 °C, DLS measurements were recorded continuously at 40 °C for 24 h. A size reduction was evident immediately after heating to 40 °C, and no changes were subsequently observed. This finding is contrary to the results observed for cylindrical PNIPAM gels, whose size displayed a slight downward trend with time, with one or two decreasing steps after time intervals lower than 1000 min for all final temperatures. These differences are surely due to the crosslinking of these PNIPAM gels.

Owing to this evidence, it seems logical to ascribe the observed dynamics to the kinetics of the coil-to-globule transition. Therefore, we designed an experiment for evaluating the final sizes of sample 3P-NP2-DEC–AC after heating continuously from 25 to 60 °C at different rates. The same sample aliquot was tested using a bath with programmable heating ramps, which allowed the aliquot to undergo the transition at different (but constant) heating rates (Figure S9). In this experiment, the aliquot was first introduced in the DLS setup, and a size measurement sequence was performed at 25 °C. Afterward, the DLS cuvette was placed inside the heating bath and subjected to a heating ramp from 25 to 60 °C at a given heating rate. Once at 60 °C, the cuvette was immediately reintroduced into the DLS setup (already at 60 °C) for a size measurement at 60 °C, followed by another one at 25 °C. In the meantime, the bath was again cooled to 25 °C. This process was repeated for 9 different heating rates. The direct heating rate reached by DLS was estimated to be 13 °C/min. Figure 8d shows the mean volume size variations (calculated from lognormal fit parameters) after heating to 60 °C as a function of the heating rate, satisfactorily fitted to a logarithmic trend. In addition, the results for selected 25/60 °C couples are collected in Figure 8e. It shows that the sizes at 25 °C are reproducible, but those at 60 °C increase as the heating rate decreases, becoming increasingly similar to those at 25 °C; that is, the size reduction upon heating became less evident. Eventually, note that, in our case, the final temperature is always the same (60 °C); thus, we highlight that the relevant factor is, in fact, the heating rate and not the final temperature, as in cylindrical PNIPAM gels. Other studies considering heating rates^44^ did not detect size differences after shrinking due to limitations in size determination. Although the origin of this heating rate behavior is beyond the scope of this work, a clue to explain this phenomenon might be found in previous studies on the LCST transition kinetics of aqueous PNIPAM solutions.^45^ They proved that the coil-to globule transition of PNIPAM follows universal nucleation and growth models. Generally, high transition rates produce more nuclei and smaller particles after growth, while low transition rates result in fewer nuclei and larger particles.

According to these results, Figure 8d stands as a preliminary calibration curve validating the use of these thermoresponsive hybrid nanospheres as heating rate nanosensors. This proves that nanospheres can reversibly disintegrate and rebuild upon heating and cooling across the LCST and that the heating rate can be directly deduced from the nanosphere size after disintegration through a logarithmic equation. Indeed, if the heating source was not an external bath but rather the magnetic nanoparticles trapped in the nanospheres subjected to alternating magnetic fields, the nanospheres, through their final size, could directly provide information on their own heating ability, in contrast to a bulk temperature sensor. Previous approaches for this purpose, i.e., for obtaining SAR information directly from the nano-object, are available using, e.g., molecular thermometers, in which the temperature can be linked to an easily detectable optical signal^39^ or to the detection of the thermal decomposition of a thermosensitive molecule.^46^ These systems are based on the measuring of the local temperature evolution with time. Since this temperature measurement is obviously not performed in adiabatic conditions, the heating rate at zero losses is calculated through an approximation, by fitting *T*(*t*) and evaluating the initial slope as

3 $$heating\;rate\;at\;zero\;losses≅{\frac{dT}{dt}|}_{t\to 0}$$

from which SAR is obtained using eq 1. By contrast, our thermoresponsive nanospheres are not thermometers (they do not measure the temperature), but they directly sense the heating rate without the use of foreign sensing molecules. These unprecedented findings open the way to a novel approach for determining the SAR, in particular, calculating the heating rate through size measurements without the use of a thermometer. Their validation as heating-rate nanosensors in a biological environment would be a natural and important next step, and the present work establishes the foundational design and characterization for such studies.

## 4 Conclusions

Miniemulsion and solvent evaporation was combined with decantation separation to fabricate colloidal suspensions of PVA–PNIPAM–PLGA hybrid magnetic thermoresponsive nanospheres, through a facile method that innovatively starts with PNIPAM as polymer. Miniemulsion was formed using the natural globalization of PNIPAM above the LCST, thus preventing the typical use of ultrasonication. Contrarily to nanospheres gathered, either directly after solvent evaporation, or in organic decants, nanospheres collected in aqueous decants were stable in water for months due to the steric effect of the PVA present on surface. Thermal analysis further unveiled that PVA and PNIPAM formed polymer blends with more/less PVA in aqueous/organic decants, respectively, in accordance with the observed good/poor colloidal stability. NPs were found to modulate the polymeric content of nanospheres, since full polymer integration to nanospheres were achieved only with the highest NP uptake, suggesting different optimal preparation quantities. Suppression of PLGA promoted wider distributions and bigger sizes, inadequate for biomedical applications. Notably, nanospheres were reversibly thermoresponsive with only a 12% of PNIPAM. Also PVA played a role in thermoresponsiveness, since nanospheres poor in PVA displayed essentially no size reduction across the LCST. Besides their excellent colloidal stability, our hybrid nanospheres were biocompatible and exhibited a satisfactory heating ability when subjected to alternating magnetic fields, demonstrating their suitability for magnetic hyperthermia. Interestingly, and because the preparation process did not involve any polymer crosslinking, the mechanism for thermally induced size change involved disintegration/rebuilding of the nanospheres, instead of the typical shrinking/swelling behavior of PNIPAM microgels. Moreover, analysis of the coil-to-globule transition dynamics allowed us to correlate the nanosphere size after disintegration with the heating rate across the LCST, thus validating the use of our hybrid nanospheres as unprecedented dual hyperthermia agents and reversible heating rate nanosensors without the use of foreign nanothermometers.

## Supplementary Material



## Notes

List of sample acronyms; TEM, magnetic and magnetothermal characterization of NP1 and NP2 nanoparticles; details on the design of preparation method; DLS characterization of PVA and PNIPAM aqueous solutions; size change reproducibility of 3P suspensions of nanospheres; DLS intensity, volume and number statistics for the PVA and PNIPAM solutions and 3P suspensions; TEM of 3P-noNP-DEC-AC; DLS intensity, volume and number distributions and statistics for 2P DEC–AC suspensions; comparison of ILP values; cell morphology after the lactate dehydrogenase (LDH) assay; DLS characterization and heating ramps to assess the aptitude of 3P nanospheres as heating rate nanosensors (PDF)
