# Supplementary material for: Uncrosslinked Thermoresponsive Hybrid Magnetic Nanospheres Directly Prepared from Poly(N‑isopropylacrylamide) that Behave as Heating Rate Nanosensors
Source: Biomacromolecules. 2025 Oct 17;26(11):8184–99. doi: 10.1021/acs.biomac.5c01603 (PMC12606644; doi:10.1021/acs.biomac.5c01603)
Supplement: Supplementary file 1 [file bm5c01603_si_001.pdf]

Uncrosslinked thermoresponsive hybrid magnetic nanospheres directly prepared from Poly(N-isopropylacrylamide) that behave as heating rate nanosensors

*María García-Maestre, Laura Cervera-Gabalda and Eva Natividad**

Instituto de Nanociencia y Materiales de Aragón (INMA), CSIC - Universidad de Zaragoza, Campus Río Ebro, María de Luna 3, 50018 Zaragoza, Spain

SUPPORTING INFORMATION

List of sample acronyms

Aqueous polymer solutions	
**Sol-PVA**	Aqueous PVA solution at concentration of 7.5 mg/mL
**Sol-PNIPAM**	Aqueous PNIPAM solution at concentrations of 5.0 mg/mL

3P: nanospheres prepared using PVA, PLGA and PNIPAM without or with different NPs	
**3P-noNP**	PVA/PLGA/PNIPAM system without magnetic nanoparticles
**3P-noNP-RAW**	3P-noNP sample before separation by decantation
**3P-noNP-DEC-AC**	3P-noNP sample after separation by decantation – aqueous decant
**3P-noNP-DEC-ORG**	3P-noNP sample after separation by decantation – organic decant
**3P-NP1**	PVA/PLGA/PNIPAM system with NP1 magnetic nanoparticles
**3P-NP1-RAW**	3P-NP1 sample before separation by decantation
**3P-NP1-DEC-AC**	3P-NP1 sample after separation by decantation – aqueous decant
**3P-NP1-DEC-ORG**	3P-NP1 sample after separation by decantation – organic decant
**3P-NP2**	PVA/PLGA/PNIPAM system with NP2 magnetic nanoparticles
**3P-NP2-RAW**	3P-NP2 sample before separation by decantation
**3P-NP2-DEC-AC**	3P-NP2 sample after separation by decantation – aqueous decant
**3P-NP2-DEC-ORG**	3P-NP2 sample after separation by decantation – organic decant

2P: nanospheres prepared using PVA and PNIPAM (replacing PLGA by PNIPAM) without or with different NPs	
**2P-noNP**	PVA/PNIPAM system without magnetic nanoparticles
**2P-noNP-DEC-AC**	2P-noNP sample after separation by decantation – aqueous decant
**2P-NP1**	PVA/PNIPAM system with NP1 magnetic nanoparticles
**2P-NP1-DEC-AC**	2P-NP1 sample after separation by decantation – aqueous decant
**2P-NP2**	PVA/PNIPAM system with NP2 magnetic nanoparticles
**2P-NP2-DEC-AC**	2P-NP2 sample after separation by decantation – aqueous decant
**2P-NP2 (100%PVA)**	The same sample than 2P-NP2 (it is assumed to be prepared with 100% PVA)
**2P-NP2 (25%PVA)**	Similar than 2P-NP2 (100%PVA), but prepared with a quarter of the PVA mass
**2P-NP2 (50%PVA)**	Similar than 2P-NP2 (100%PVA), but prepared with half the PVA mass
**2P-NP2 (200%PVA)**	Similar than 2P-NP2 (100%PVA), but prepared with twice the PVA mass
**2P-NP2 (400%PVA)**	Similar than 2P-NP2 (100%PVA), but prepared with four times the PVA mass

TEM, magnetic and magnetothermal characterization of NP1 and NP2 nanoparticles

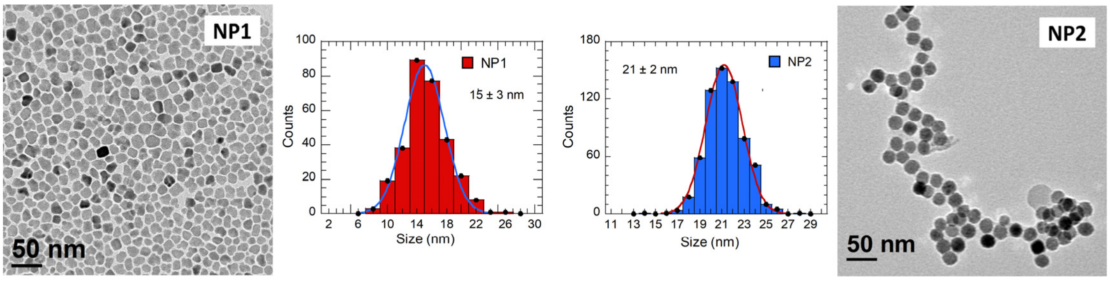

Figure S1. TEM micrographs of NP1 and NP2 nanoparticles, together with histograms obtained by measuring the size of 301 and 648 NPs, respectively. Lines are fits to normal distributions, from which mean values and standard deviations were calculated, namely, 15 ± 3 nm and 21 ± 2 nm for NP1 and NP2, respectively.

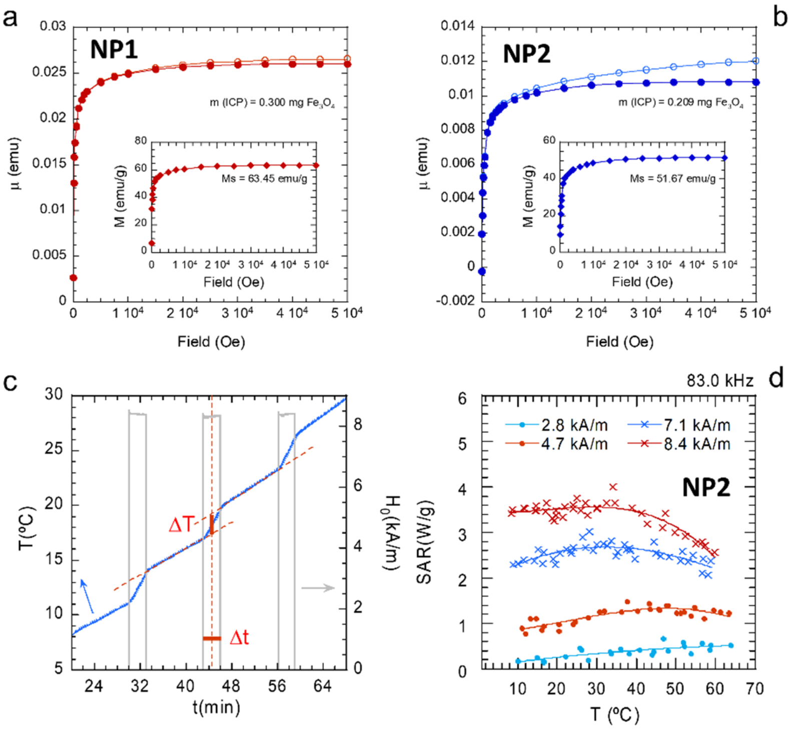

Figure S2. a-b: Magnetization measurements against static field, $M(H)$, at 300K of NP1 and NP2 nanoparticles, respectively. Open circles: total magnetic moment, μ (emu). Full circles: ferromagnetic contribution to μ, calculated subtracting linear μ (H) trends assigned to para/diamagnetic contributions. Full diamonds: M (emu/g), obtained dividing ferromagnetic μ (H) by the mass of Fe_3O_4 derived by ICP-OES analysis. $M(H)$ allowed calculating saturation magnetization, M_S, which was used to quantify the mass of magnetic material trapped in the nanospheres, from new μ (H) measurements of freeze-dried suspensions. c: Typical $T(t)$ characteristics together with calculations of ΔT and Δt for one heating pulse. d: SAR(T) trends of dry NP2 nanoparticles for several magnetic field amplitudes, H_0, measured at an ac field frequency of 83.0 kHz and fitted to polynomial functions to guide the eyes.

Details on the design of preparation method

We started from one method aimed at obtaining PLGA/PVA nanospheres[1,2] loaded with NPs, which consisted of five steps: 1) preparation of organic and aqueous solutions; 2) emulsion preparation by mixing both solutions and magnetic stirring; 3) miniemulsion preparation by tip ultrasonication of this emulsion; 4) organic solvent evaporation by stirring; and 5) elimination of excess PVA by dialysis. Preliminary tests were performed following this initial method, substituting part of the PLGA with PNIPAM (the polymer, not the NIPAM monomer). However, two problems arose related to steps 3/5.

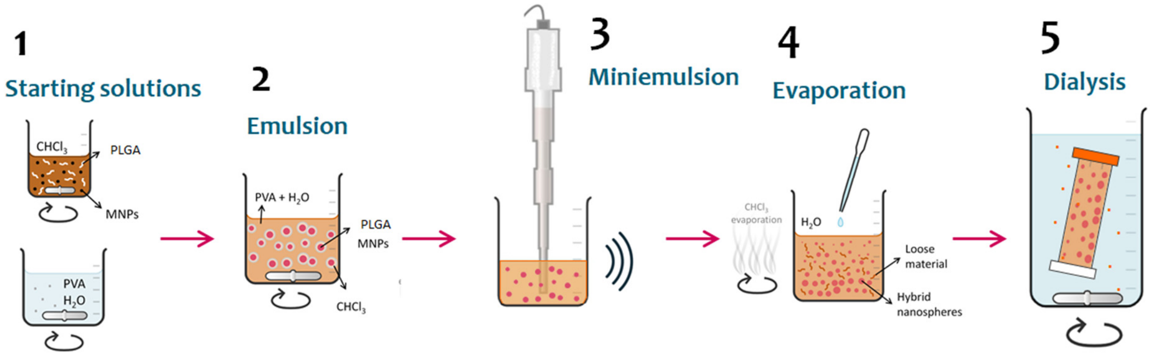

Scheme S1. Preparation process for obtaining PLGA/PVA nanospheres loaded with magnetic NPs.

The first issue was that a too dense solution was obtained during step 3, leading to undesirable macroscopic segregation upon solvent evaporation. Notably, given that PNIPAM stayed below its LCST during the process, it remained soluble in both water and chloroform, behaving differently from PLGA, which is water insoluble. To overcome this issue, we opted to eliminate the ultrasonication stage and take advantage of the potential of PNIPAM to create its own miniemulsion above the LCST owing to its coil-to-globule transition. Accordingly, the process was modified to be fully carried out on a hot plate at 70°C, with a solution temperature of *ca.* 60°C, to ensure that PNIPAM, insoluble in water but soluble in chloroform at this temperature, could form small dispersed phase domains during chloroform evaporation.

The second issue was related to step 5. When applied to PLGA/PVA nanospheres, dialysis using 300 kDa Float-A-Lyzer membranes was effective at removing excess PVA. However, this process was ineffective at eliminating all the apparent loose material in the PNIPAM/PLGA/PVA nanospheres, even when using 1000 kDa membranes. This loose material not only acted as an impurity but also hindered the correct thermoresponsive characterization of the nanospheres. Ultracentrifugation (10000-15000 rpm) was also tested for purification, but the results were inconclusive after several stages of supernatant/residue separation. Finally, step 5 was replaced by a process of separation by decantation using water and $CHCl_3$. Given that PNIPAM, which was suspected to be responsible for dialysis failure, tends to solubilize preferentially in chloroform, this decantation aimed at separating this polymer from PVA-covered nanospheres, which were supposed to remain suspended in water.

[1] M. Urban, A. Musyanovych and K. Landfester, *Macromol. Chem. Phys.* 210 (**2009**) 961-970. https://doi.org/10.1002/macp.200900071
[2] I. Andreu, E. Natividad, L. Solozábal and O. Roubeau, *ACS Nano* 9 (**2015**) 1408-1419. https://doi.org/10.1021/nn505781f

DLS characterization of PVA and PNIPAM aqueous solutions

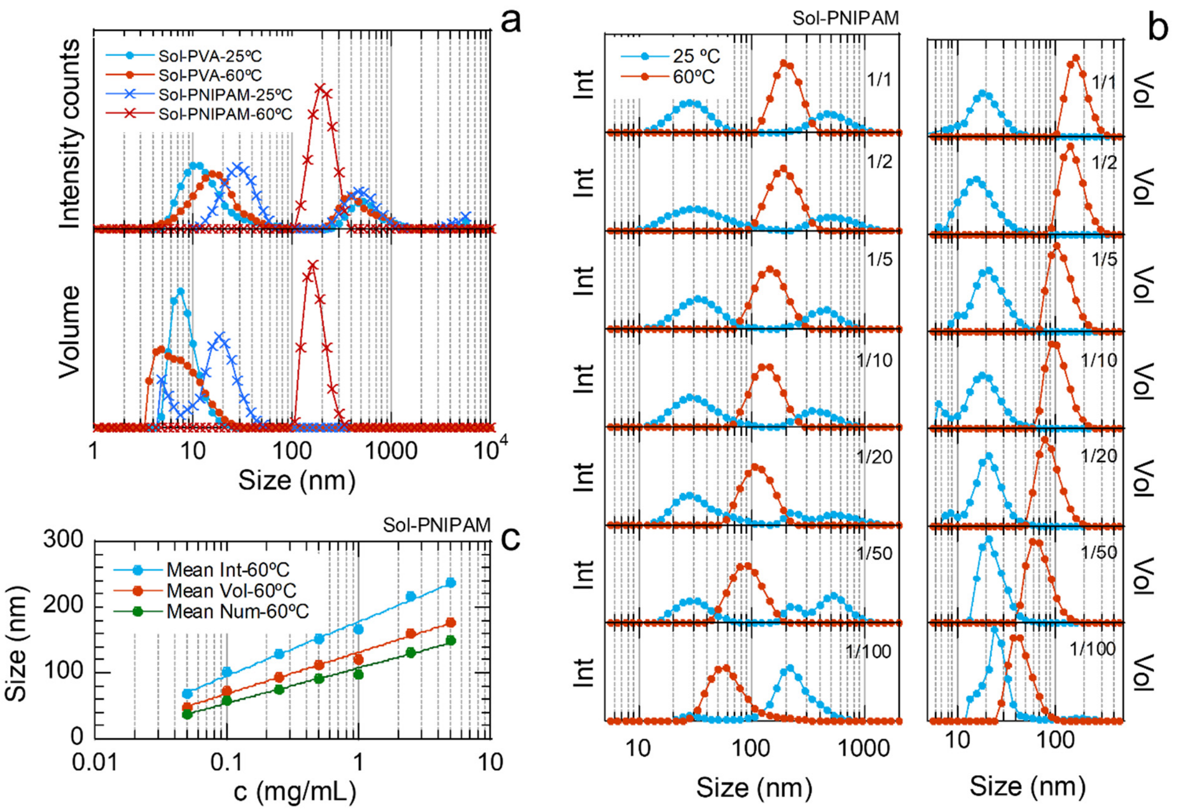

Figure S3. DLS characterization of PVA and PNIPAM aqueous solutions. The lines in the DLS distributions guide the eyes. a: DLS intensity and volume distributions at 25 and 60°C. b: Evolution of the DLS intensity and volume distributions with decreasing solution concentration. c: Mean values of intensity, volume and number distributions of PNIPAM solution at 60°C at the studied concentrations together with logarithmic fit y = a + b*log(x), where a = 178/132/108 and b = 82/63/54 for intensity/volume/number, respectively.

Size change reproducibility of 3P suspensions of nanospheres

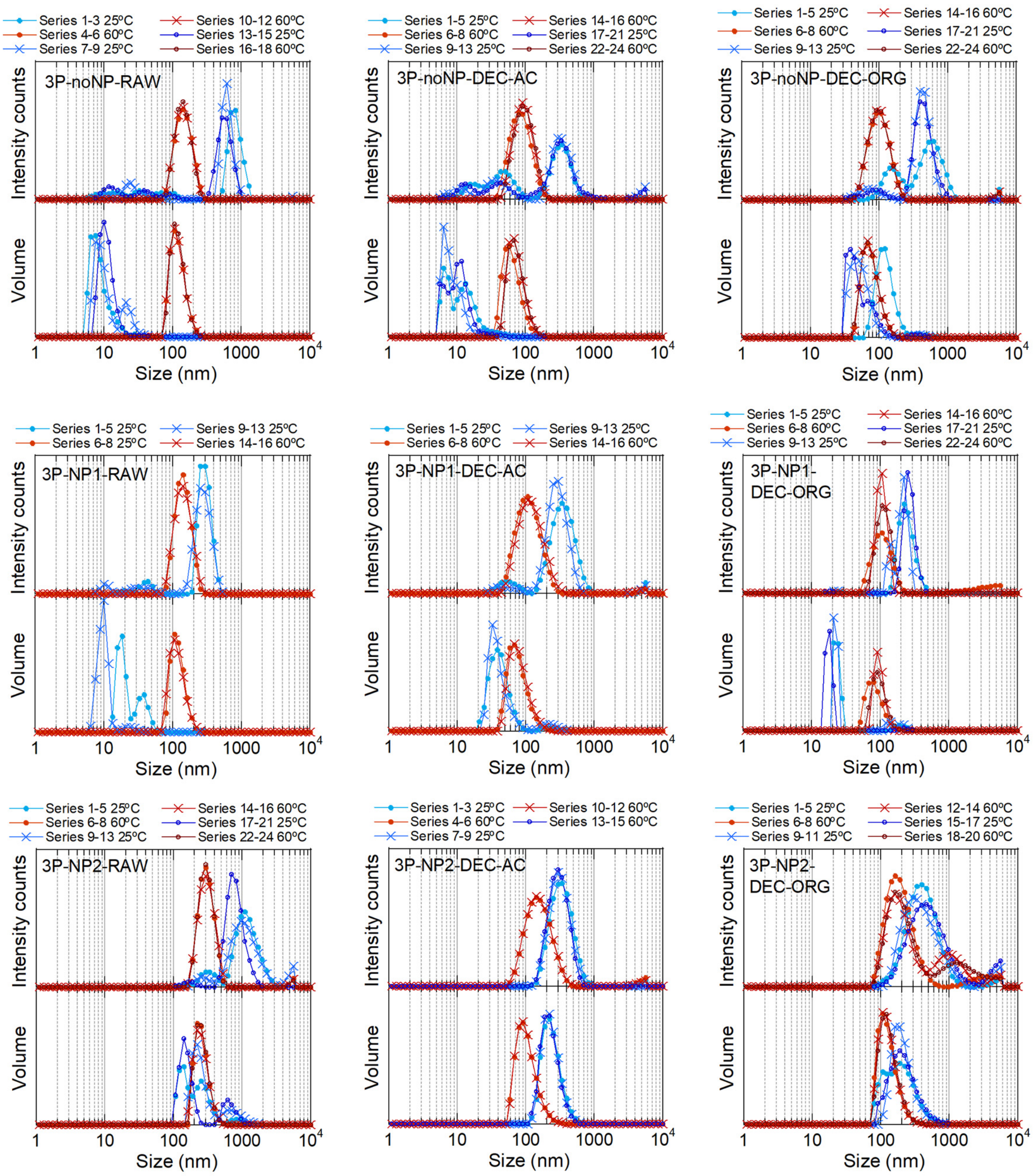

Figure S4. Alternate DLS measurements at 25°C (series in blue, below the LCST of PNIPAM) and 60°C (series in red, well above the LCST of PNIPAM) of all 3P suspensions of nanospheres. DLS measurements were conducted in series of 5 measurements with a rest of 10 seconds. Typically, between 3 and 5 of these series were recorded and averaged for each temperature. Plots collect these average distributions, which reflect the good reproducibility and reversibility of the suspensions upon heating/cooling. Note that the number of the series is correlative, denoting the heating and cooling steps undergone by each sample. In particular, sizes at 60°C are extraordinarily reproducible for all samples. At 25°C, DEC-AC samples display the best reproducibility, especially 3P-NP2-DEC-AC, which has no loose material. This sample was subjected to: a first reassembly after preparation (series 1-3, at 25°C), a first disintegration (series 4-6, at 60°C), a second reassembly (series 7-9, at 25°C), a second disintegration (series 10-12, at 60°C) and a third reassembly (series 13-15, at 25°C). Series at 25°C (1-3, 7-9 and 13-15) overlap perfectly and so do series at 60°C (4-5, 10-12).

DLS intensity, volume and number statistics for the PVA and PNIPAM solutions and 3P suspensions

Sample	T (°C)	State	Intensity				Volume				Number			
			d_{LN} (nm)	σ_{LN} (nm)	d_m (nm)	σ (nm)	d_{LN} (nm)	σ_{LN} (nm)	d_m (nm)	σ (nm)	d_{LN} (nm)	σ_{LN} (nm)	d_m (nm)	σ (nm)
**Sol-PVA**	25	-	14	0.42	15	7	8	0.28	9	3	7	0.19	7	1
	60	-	20	0.48	22	13	9	0.50	10	6	4	0.18	4	1
**Sol-PNIPAM**	25	-	33	0.37	35	14	22	0.38	23	10	13	0.39	14	6
	60	-	216	0.27	224	64	173	0.23	178	43	148	0.18	150	27
**3P-noNP**	25	RAW	693	0.25	715	189	10	0.28	10	3	8	0.22	8	2
		DEC-AC	368	0.33	388	137	10	0.42	11	5	6	0.15	6	1
		DEC-ORG	503	0.32	530	185	112	0.45	124	64	98	0.28	102	31
	60	RAW	154	0.29	160	49	120	0.24	124	32	101	0.19	102	19
		DEC-AC	102	0.35	108	41	72	0.28	75	22	58	0.21	59	13
		DEC-ORG	111	0.35	118	46	76	0.34	80	30	62	0.26	64	17
**3P-NP1**	25	RAW	300	0.24	309	76	15	0.40	16	7	15	0.46	17	9
		DEC-AC	355	0.37	380	155	40	0.28	42	12	32	0.21	33	7
		DEC-ORG	249	0.22	256	59	22	0.15	22	3	22	0.12	22	3
	60	RAW	151	0.28	157	47	119	0.24	123	31	100	0.19	102	20
		DEC-AC	131	0.42	143	68	79	0.31	83	27	61	0.22	62	14
		DEC-ORG	113	0.25	116	30	95	0.23	97	23	84	0.20	86	18
**3P-NP2**	25	RAW	1105	0.41	1200	553	215	0.40	232	104	163	0.32	171	59
		DEC-AC	351	0.36	374	147	241	0.29	251	79	188	0.23	193	46
		DEC-ORG	541	0.56	634	454	228	0.43	249	122	142	0.34	151	57
	60	RAW	319	0.27	331	93	258	0.22	264	61	222	0.17	225	38
		DEC-AC	181	0.44	199	101	104	0.31	109	36	79	0.21	81	17
		DEC-ORG	210	0.43	230	112	128	0.29	134	40	102	0.20	104	21

Table S1. DLS intensity, volume and number statistics at 25 and 60°C for the PVA and PNIPAM solutions and 3P suspensions, considering only the *main* peak in each case. d_{LN} and σ_{LN} are the parameters obtained from the lognormal fit of the distributions, and d_m and σ are the calculated mean values and standard deviations, respectively.

TEM of 3P-noNP-DEC-AC

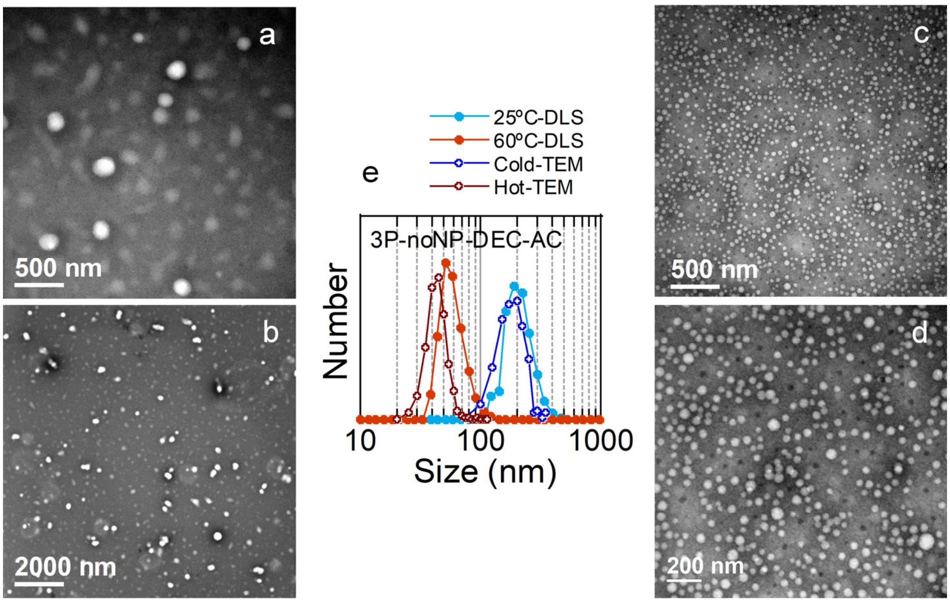

Figure S5. Negatively stained TEM micrographs of 3P-noNP-DEC-AC left dry on carbon-coated Cu grids at room temperature (cold-TEM: a, b) and on hot plate at 70°C (hot-TEM: c, d). Note that images (a) and (c) have the same magnification. (e) DLS number distributions of 3P-noNP-DEC-AC at 25 and 60°C, together with TEM distributions obtained from cold-TEM and hot-TEM micrographs.

DLS intensity, volume and number distributions and statistics for 2P DEC-AC suspensions

Sample	%PVA	T (°C)	Intensity				Volume				Number			
			d_{LN} (nm)	σ_{LN} (nm)	d_m (nm)	σ (nm)	d_{LN} (nm)	σ_{LN} (nm)	d_m (nm)	σ (nm)	d_{LN} (nm)	σ_{LN} (nm)	d_m (nm)	σ (nm)
**2P-noNP**	100	25	313	0.48	352	202	8	0.23	9	2	7	0.18	8	1
		60	145	0.41	158	73	90	0.32	94	32	68	0.24	70	17
**2P-NP1**	100	25	578	0.67	723	679	11	0.43	12	6	8	0.25	8	2
		60	159	0.40	172	78	100	0.30	105	34	77	0.22	79	18
**2P-NP2**	25	25	391	0.66	485	440	130	0.44	143	72	73	0.35	78	30
		60	202	0.42	221	107	121	0.30	127	41	94	0.22	96	22
	50	25	218	0.46	242	132	121	0.34	128	47	94	0.20	96	20
		60	162	0.36	173	69	111	0.28	116	34	89	0.20	91	19
	100	25	619	0.52	709	455	114	0.52	131	84	83	0.42	90	43
		60	418	0.60	500	392	58	0.29	61	19	48	0.23	49	12
	200	25	305	0.44	336	169	175	0.33	185	66	130	0.23	134	32
		60	161	0.39	174	76	103	0.30	107	34	80	0.21	82	18
	400	25	384	0.49	433	253	195	0.37	208	84	135	0.28	140	41
		60	252	0.49	284	167	124	0.38	134	57	85	0.28	88	26

Table S2. DLS intensity, volume and number statistics at 25 and 60°C for 2P DEC-AC suspensions, considering only the *main* peak in each case. d_{LN} and σ_{LN} are the parameters obtained from the lognormal fit of the distributions, and d_m and σ are the calculated mean values and standard deviations, respectively.

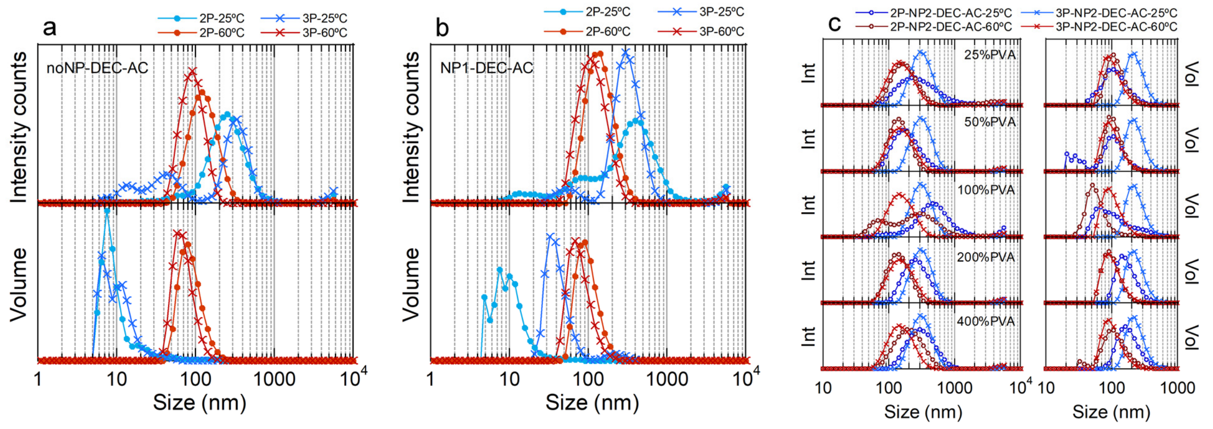

Figure S6. DLS intensity and volume distributions of the 2P DEC-AC samples and comparison with 3P analogues. a: noNP. b: NP1. c: NP2. For the latter, the 2P % mass of PVA ranged between 25 and 400%, with 100% being the nominal value. The lines guide the eyes.

Comparison of ILP values

Ref.	Sample	Iron compound	Medium	f (kHz)	H_0 (kA/m)	SAR (W/g*)	SAR (W/g_{Fe})	ILP ($nH{\cdot}m^2/kg_{Fe}$)
**This work**	Dry NP2	Fe_3O_4 (nanospheres)	Dry (oleic acid)	83	8.4	3.6	5.0	0.9
	3P-NP2-DEC-AC	Fe_3O_4 (nanospheres)	Freeze-dried (PNIPAM/PLGA/PVA)	83	8.4	5.2	7.2	1.2
**2010**[3]	1	Fe^0 (nanocubes)	Mesitylene	300	52.8	1690	1690	2.0
**2012**[4]	NF(s,rh2/1,12)	Fe_2O_3 (nanoflowers)	Water	700	21.5	1944	2846	8.6
**2015**[5]	S-SURF	Fe_3O_4 (nanocubes)	Dry (oleic acid)	111	3	0.4	0.6	0.6
	L-HEX	Fe_3O_4 (nanocubes)	Hexane	111	3	2	2.8	2.8
	S-PLGA	Fe_3O_4 (nanocubes)	Freeze-dried (PLGA/PVA)	111	3	0.8	1.1	1.1
**2021**[6]	PAA-IONFs 2nd feeding step	Fe_2O_3/Fe_3O_4 (nanoflowers)	Water	488	24.5	2426	2426	8.3

Table S3. Comparison of ILP values of different nanoparticles, arrangements and dispersive media: reference to the work; specific sample; iron compound present and shape of NPs; dispersive medium; frequency (*f*) and field amplitude (H_0) used for the measurements; SAR as appears in the reference; SAR referred to mass of Fe; and ILP values referred to mass of Fe.

ILP is a heating ability indicator that aims to remove the influence of using more or less powerful applied magnetic fields in SAR determination. It is calculated as ILP = $10^3 \cdot SAR/(f \cdot H_0^2)$, with SAR in W/g, *f* in kHz and H_0 in kA/m. Additionally, it is a good practice to compare SAR (ILP) values for magnetic materials of the same mass; for example, some authors use the mass of the whole magnetic compound, while others use only the mass of iron, especially when the compound is unknown or there is a mixture of several compounds. Considering that the ILP refers to the mass of iron (Table S3), we can compare our PVA/PLGA/PNIPAM/NP (ILP = 1.2 $nH{\cdot}m^2/kg_{Fe}$) and our previous PVA/PLGA/NP nanospheres[5] (S-PLGA, ILP = 1.1 $nH{\cdot}m^2/kg_{Fe}$). We conclude that both of these agents have similar ILP values, but the latter results in greater improvement from free NPs to NPs integrated on nanospheres (factor 2 against factor 1.5). This could be explained by the kind of NPs used since PVA/PLGA nanospheres contain nanocubes (S-SURF) that can acquire more advantageous arrangements on the surface. In addition, nanocubes can reach 2.8 $nH{\cdot}m^2/kg_{Fe}$ under most advantageous conditions (L-HEX, measured in liquid, thus indicating the possibility of self- or field-induced assembly). In summary, the heating ability of the dried 3P-NP2-DEC-AC nanospheres is good, but there is still room for improvement. The NP arrangement and concentration on the nanosphere surface could be optimized (a too high concentration can degrade SAR), or another kind of magnetic nanoparticle could be used, such as nanoflowers or special multicore nanoparticle, to reach ILP values of 8.3 – 8.6 $nH{\cdot}m^2/kg_{Fe}$ (see Table S3).

[3] B. Mehdaoui, A. Meffre, L.-M. Lacroix, *et al.*, *J. Magn. Magn. Mater.* 322 (**2010**) L49–L52; 10.1016/j.jmmm.2010.05.012
[4] P. Hugounenq, M. Levy, D. Alloyeau, *et al.*, *J. Phys. Chem. C* 116 (**2012**) 15702−15712; 10.1021/jp3025478
[5] I. Andreu, E. Natividad, L. Solozábal and O. Roubeau, *ACS Nano* 9 (**2015**) 1408–1419; 10.1021/nn505781f
[6] L. Storozhuk, M. O. Besenhard, S. Mourdikoudis, *et al.*, *ACS Appl. Mater. Interfaces* 13 (**2021**) 45870−45880; 10.1021/acsami.1c12323

Cell morphology after the Lactate Dehydrogenase (LDH) assay

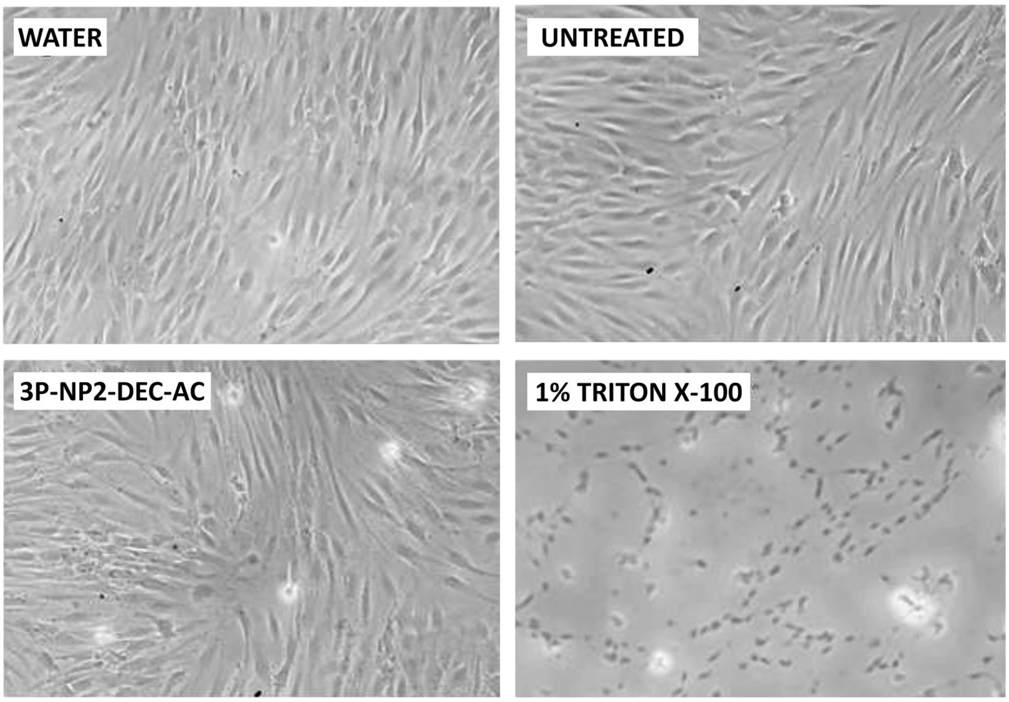

Figure S7. Cell morphology after the LDH assay. Neither the cells treated with the water used for sample preparation, nor those treated with 3P-NP2-DEC-AC nanospheres, showed significant differences from the untreated cells (negative control), displaying a fibroblastic morphology typical of the cell type used. However, all cells treated with Triton X-100 1% were dead (positive control). This supports that 3P-NP2-DEC-AC nanospheres are not cytotoxic at a concentration of 0.5 mg/mL after 24 hours of treatment.

DLS characterization and heating ramps to assess the aptitude of 3P nanospheres as heating rate nanosensors

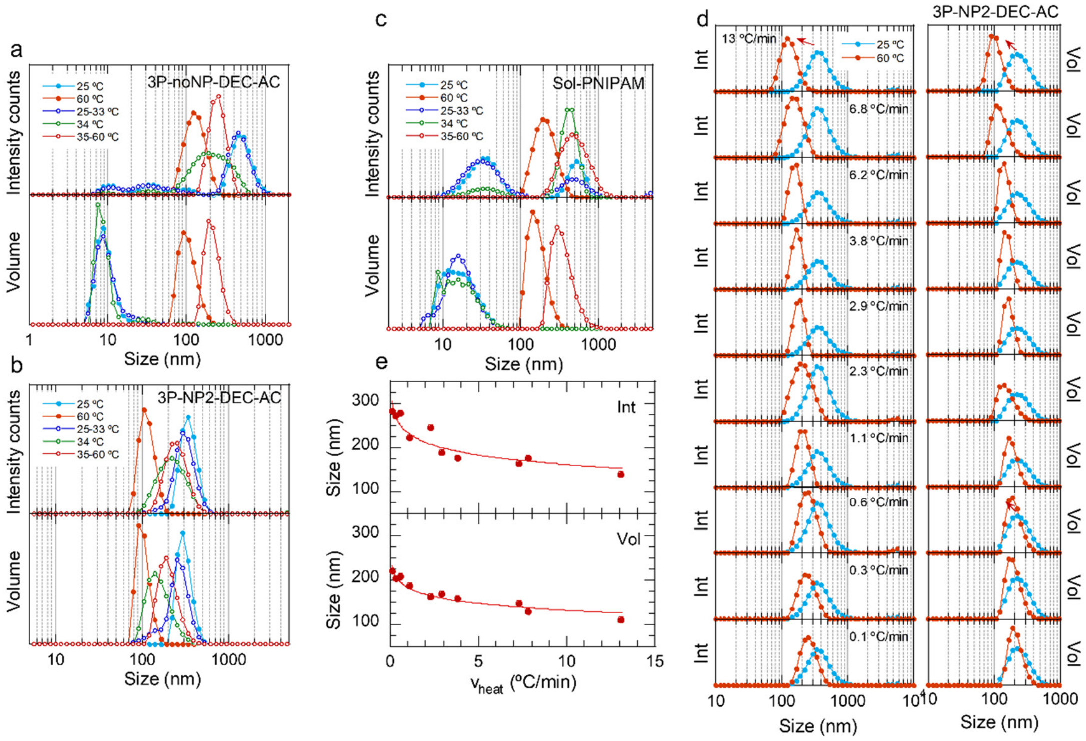

Figure S8. **a-c:** DLS intensity and volume distributions of 3P-noNP-DEC-AC (a), 3P-NP2-DEC-AC (b) and PNIPAM solution (c) after direct heating between 25 and 60°C (full symbols) and stepped heating ramps following the sequence 25-30-31-32-33-34-35-36-38-40-60°C (open symbols). An average is shown for the measurements between 25 and 33°C and between 35 and 60°C since they were identical within each interval. d: DLS intensity and volume distributions of 3P-NP2-DEC-AC nanospheres at 25°C and after heating to 60°C with the help of a bath with programmable heating ramps ranging between 6.8 and 0.1°C/min (a 13°C/min rate is achieved only by DLS). The lines in all the DLS distributions guide the eyes. e: mean size variations in intensity and volume at 60°C (calculated by lognormal fitting distributions in (d)) as a function of the heating rate. Lines are fits to logarithmic trends, y = a - b*log(x), where a = 234/183 and b = 72.0/50.3 for intensity/volume.

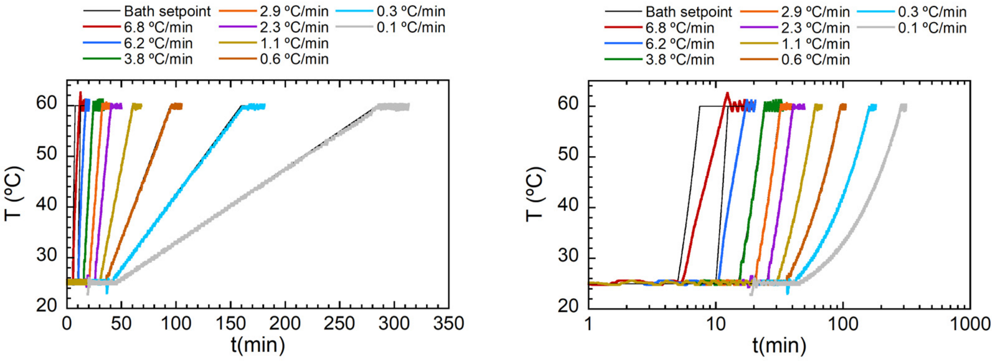

Figure S9. Temperature versus time heating ramps applied to 3P-NP2-DEC-AC by means of a bath with programmable ramps (same plot with different time scales). Black lines are the setpoint temperatures established in each ramp. Note that, for the two fastest ramps, the bath failed to achieve the setpoint temperature, and the heating rates were calculated in the 25 – 40°C temperature intervals, in which rates were constant and the LCST transition took place. Accordingly, 6.8°C/min was the fastest heating rate achieved with the bath. Directly with DLS, a maximum rate of 13°C/min was obtained.
